# *GLK1* plays a positive role in regulating plant tolerance to alkali stress and salt-alkali mixed stress

**DOI:** 10.3389/fpls.2026.1901920

**Published:** 2026-09-18

**Authors:** Qinyan Bao, Lin Jiang, Zhaozhu Wen, Wenxuan Du, Jiangqi Wen, Kirankumar S. Mysore, Juan Sun, Zeng-Yu Wang

**Affiliations:** 1 College of Pastoral Agriculture Science and Technology, Lanzhou University, Lanzhou, China; 2 Shandong Key Laboratory for Germplasm Innovation of Saline-alkaline Tolerant Grasses and Trees, College of Grassland Science, Qingdao Agricultural University, Qingdao, China; 3 School of Medical, Molecular and Forensic Sciences, Murdoch University, Perth, WA, Australia; 4 Institute for Agricultural Biosciences, Oklahoma State University, Ardmore, OK, United States

**Keywords:** alkali and salt–alkali mixed stress, GLK1, *Medicago sativa*, *Medicago truncatula*, Y1H assay

## Abstract

The *Golden2-like 1* (*GLK1*) gene regulates photosynthesis and is associated with drought tolerance and cold tolerance. However, no report has determined the role of *GLK1* in alkali tolerance. In this study, we found that *MsGLK1* responds to alkali and salt–alkali mixed stresses in alfalfa (*Medicago sativa*) and carried out detailed functional analysis of *GLK1* in the model plants *Arabidopsis* and *Medicago truncatula*. In *Arabidopsis*, the *atglk1/2* double mutant exhibited reduced tolerance to these two stress treatments compared to the wild type. Conversely, overexpression of *MsGLK1* in *Arabidopsis* displayed enhanced tolerance under the same treatments. To further study the mechanism underlying response to these stresses, we employed the *mtglk1* mutant in *M. truncatula*, a close relative of alfalfa. The *mtglk1* mutant suffered severe damage when subjected to the two stresses. Consistent with phenotypic and physiological analyses, 1092 and 999 differentially expressed genes (DEGs) were identified under alkali and salt–alkali mixed stress, respectively. Our results showed that *GLK1* plays an important role in regulating tolerance to alkali and salt–alkali mixed stress. A yeast one-hybrid assay revealed that MtGLK1 directly binds to the promoter of the *MtMYB*-*HB*-*like* gene.

## 1 Introduction

Soil salinization and alkalization are major threats to sustainable agriculture (Aouz et al., 2023). The global cultivated land area is approximately 1.5×10^9^ ha, of which 23% is saline and 37% is alkaline (Shi and Wang, 2005). It has been predicted that 50% of the world’s soil will be affected by salinization by the end of 2050 (Khan et al., 2023). Soil salinization and alkalization often occur simultaneously under natural conditions (Fang et al., 2021), however, it is recognized that their effects and mechanisms on plants differ (Shi and Sheng, 2005; Wang et al., 2012; Guo et al., 2015; Wan et al., 2024). Salinization is caused by neutral salts (mainly NaCl and Na_2_SO_4_). Excessive sodium ions hinder plant metabolism and reduce soil water potential, causing physiological drought and osmotic stress (Zhu, 2001; Wong et al., 2006). Alkalization is caused by basic salts (mainly NaHCO_3_ and Na_2_CO_3_) (Wang et al., 2017; Abebe and Tu, 2024). To date, a large number of studies have focused on determining the effects of salt on plants and the responses of plants to salt stress at the physiological and molecular levels (Anower et al., 2013; Luo et al., 2023; Zhang et al., 2025), but there are relatively few reports on alkali stress (Sun et al., 2020; Yuan et al., 2022). In addition to the toxicity of sodium ions, alkalization induces high pH damage (Jia et al., 2019). A high pH can destroy plant roots, precipitate trace metal ions and weaken nutrient absorption, utilization and recycling (Wang et al., 2011, 2022). The inhibition of photosynthesis by alkalization is more severe than that by neutral salts (Zhang et al., 2020a, 2024). Therefore, plant responses to alkali stress and salt–alkali mixed stress should be given the same level of attention as salt stress.

Transcription factors (TFs) regulate numerous biological processes and play an important role in responses to environmental stimuli. TFs involved in stress responses, including CBF/DREB, WRKY, AP2, MYBs, NAC and ABF/AREB, have been widely studied (Yamaguchi-Shinozaki and Shinozaki, 2006; Xu et al., 2011; Puranik et al., 2012; Jiang et al., 2017). TFs can sense changes in the upstream environment and regulate gene expression to address environmental challenges, such as salt stress, by binding to the cis-acting elements of downstream genes (Yoo et al., 2005; Hu et al., 2006; Orellana et al., 2010; Wu et al., 2019; Zhang et al., 2020b; Huang et al., 2022; Bhat et al., 2024). The Golden2-like (GLK) TF belongs to the GARP superfamily and was first reported in corn (Jenkins, 1926; Hall et al., 1998). *GLK* genes are conserved across plant lineages and participates in the transcriptional activation of chloroplast development and the regulation of nuclear photosynthesis-related genes (Rossini et al., 2001; Yasumura et al., 2005; Waters et al., 2009; Powell et al., 2012; Nguyen et al., 2014). In *Arabidopsis*, *GLK1* and *GLK2* redundantly regulate chloroplast development and *atglk1/2* results in a pale green leaves phenotype (Fitter et al., 2002). *GLK* genes also play important roles in defense responses (Murmu et al., 2013; Townsend et al., 2018; Li et al., 2022). Additionally, *GLK* genes are involved in abiotic stress tolerance. The chimeric repressors of *GLKs* regulate stomatal movement and confer ozone tolerance (Nagatoshi et al., 2016). *GLKs* regulate drought tolerance by adjusting stomatal opening and closing through abscisic acid (Liu et al., 2018, 2020; Li et al., 2024); *GLK1* also regulates cold stress response (Liu et al., 2021). To date, there has no reports on the involvement of *GLK1* on alkali stress tolerance.

Alfalfa (*Medicago sativa*) is one of the most important perennial leguminous forage crops worldwide (Liu et al., 2013; Luo et al., 2022). It is widely cultivated due to its high yield, nutritional value and good ecological adaptability (Liu et al., 2016; Chen et al., 2020). Severe salt and alkali stresses reduce alfalfa yield and quality (Bhattarai et al., 2020). The functional characterization of genes associated with salt and alkali stresses response in alfalfa is of great importance.

*Medicago truncatula*, a close relative of alfalfa, is regarded as the key model legume species because of its small genome, diploidy, self-pollination and short life cycle. The generation of a *Tnt1* retrotransposon-tagged mutant population (Tadege et al., 2005; Tadege et al., 2008) and genome sequencing (Young et al., 2011; Kaur et al., 2021) have facilitated the utilization of *M*. *truncatula* mutants (Zhou et al., 2014; Sun et al., 2021; Zhao et al., 2021). Many genes responsive to abiotic stress have been identified in *M. truncatula* using mutants (Kim and Nam, 2013; Nguyen et al., 2013; Lu et al., 2023; Du et al., 2024; Safavi-Rizi et al., 2025). Gene functions are often characterized in *M. truncatula* before they are utilized in alfalfa (Zhou et al., 2011; Gou et al., 2017; Du et al., 2021; Min et al., 2022).

In this study, we identified *MsGLK1* as a potential gene responsive to alkali stress. Using *Arabidopsis atglk* mutants and *MsGLK1* overexpression lines, we demonstrated the involvement of *GLK1* in plant responses to alkali stresses, based on its effects on seed germination, root growth, soil-based plant phenotypes and key physiological parameters. Furthermore, *mtglk1* mutant in *M. truncatula* was characterized in detail and used to reveal regulatory networks. Our results reveal that *GLK1* positively regulates alkali and salt–alkali mixed stress tolerance by directly activating the *MYB*-*HB*-*like* gene.

## 2 Materials and methods

### 2.1 Plant materials and growth conditions

*Arabidopsis* mutants *atglk1* (N9805), at*glk2* (N9806) and *atglk1/2* (N9807) were obtained from the Arabidopsis Biological Resource Centre (ABRC, https://abrc.osu.edu/). Seeds were sterilized with ethanol and sodium hypochlorite, plated on ½ Murashige and Skoog (MS) medium and stratified at 4 °C for 2 days, germination and root growth experiments were conducted in a growth chamber with light intensity of 150 μmol/m^2^/s (16 h/8 h day/night) at 24°C day/22°C night temperature.

*M. truncatula* ecological type R108 was used as the wild type for comparison with the mutants (Tang et al., 2014). The *mtglk1* (NF20408) mutant was isolated from the mutant library tagged with the *Tnt1* retrotransposon. *Arabidopsis* and *M. truncatula* plants were grown in a greenhouse at 24 °C with 60–70% relative humidity under a 16-/8-h light/dark cycle.

### 2.2 Overexpression vector construction and generation of transgenic *Arabidopsis*

The CDS of *MsGLK1* was amplified from leaf cDNA of alfalfa (Regen SY4D), where *MsGLK1* exhibits the highest expression with the primer pFGC-MsGLK1. After transformation and Sanger sequencing of positive clones, two distinct haplotypes were detected. The more abundant transcript isoform was cloned into the BamH I-digested pFGC plasmid (p35S:eYFP) via homologous recombination to generate the p35S:MsGLK1-eYFP vector. *Agrobacterium tumefaciens* strain EHA105 carrying the p35S:MsGLK1-eYFP vector was used to transform *Arabidopsis* ecotype Columbia via floral dip transformation (Clough and Bent, 1998). Transformed *Arabidopsis* seeds were screened on ½ MS medium containing 7.5 mg/L phosphinothricin (Miller et al., 2015). Transgenic plants were confirmed using PCR (using primers MsGLK1 and Bar) and RT-qPCR analyses (using primer pair qPCR-MsGLK1). T3 homozygous lines were used for the study. All primers used in this study are listed in **Supplementary Table 1**.

After cloning and Sanger sequencing of positive transformants, two distinct haplotypes were identified. The more abundant transcript isoform was selected for all downstream functional assays.

### 2.3 Salt, alkali and salt–alkali mixed stress treatments

To determine the effects of salt, alkali and salt–alkali mixed stresses on germination, the seeds of *Arabidopsis atglk* mutant were sterilized and placed on ½ MS medium supplemented with 150 mM NaCl (salt stress), 20 mM NaHCO_3_ (alkali stress), and 85 mM NaCl + 15 mM NaHCO_3_ (salt-alkali mixed stress), respectively. Thirty seeds of each genotype were subjected to each treatment, with three independent experimental replicates. Seeds were scored as germinated once the radicle tip protruded the seed coat and extended to a length of 1 mm. The germination rate was recorded daily for one week.

To assess post-germination stress tolerance, 7-day-old *Arabidopsis* WT, *MsGLK1*-overexpression (OE) and *atglk* mutant seedlings were transplanted to ½ MS medium containing 150 mM NaCl, 5 mM NaHCO_3_, and 50 mM NaCl + 3 mM NaHCO_3_, respectively. After vertical cultivation for 15 days, root length and fresh weight were measured. For whole-plant stress phenotyping in soil, 21-day-old plants were subjected to treatments by drenching with 500 mL of 300 mM NaCl, 100 mM NaHCO_3_, and 200 mM NaCl + 100 mM NaHCO_3_ solutions, respectively, once every three days.

To determine the effects of salt, alkali and salt–alkali mixed stresses on *mtglk1* mutant in *M. truncatula*, seeds of WT and *mtglk1* were germinated, stratified for 2 days, then transferred to vermiculite. One-month-old plants were then subjected to treatments by drenching with 500 mL of 300 mM NaCl, 100 mM NaHCO_3_, and 200 mM NaCl + 100 mM NaHCO_3_, respectively, once every three days.

### 2.4 Measurement of physiological parameters

For *Arabidopsis*, 0.1-g leaf samples were collected from WT and OE seedlings before and after 6 days of stress treatment. The activities of catalase (CAT) and superoxide dismutase (SOD), as well as chlorophyll content were measured using kits (corresponding to CAT-1-Y, SOD-1-Y and CPL-1G, respectively; Suzhou Comin Biology, China). All assays were performed strictly following the standard protocols provided by the manufacturer. Each treatment comprised three biological replicates.

For *M. truncatula*, chlorophyll contents in the leaves of WT and *mtglk1* mutant were measured using the Solarbio biochemical test kit (Cat.NO BC0995, Solarbio Life Sciences, Beijing). 0.1-g leaf samples were collected from WT and *mtglk1* mutant seedlings before and after 3 days of stress treatment. The activities of SOD and peroxidase (POD) and the level of malondialdehyde (MDA) were determined using corresponding Solarbio Assay kits (BC5165, BC0025 and BC0095, respectively; Solarbio Life Sciences, Beijing, China). All assays were performed strictly following the standard protocols provided by the manufacturer. Each treatment comprised three biological replicates.

### 2.5 RT-qPCR analysis

Total RNA was extracted from the sample using a Trizol kit (Cat NO. R401, Vazyme). Single-stranded cDNA was reverse transcribed from 1 μg RNA using a HiScript III RT SuperMix for RT-qPCR (+gDNA wiper) Kit (Cat NO. R323, Vazyme). RT-qPCR was completed using a ChamQ SYBR Color qPCR Master Mix Kit (Cat NO. Q411, Vazyme) on the CFX96 Touch Real-Time PCR Detection System (Bio-Rad, Los Angeles, CA, USA). The actin gene was used as the reference gene (Liu et al., 2016) and the relative expression level was calculated using the 2^−ΔΔCt^ method. The primers used for RT-qPCR were designed using Primer 5.0. The primer information is provided in **Supplementary Table 1**.

### 2.6 RNA-seq assay

One-month-old plants of *M. truncatula* grown in vermiculite were treated with salt, alkali and salt–alkali mixed solutions. Leaves were collected from WT and *mtglk1* plants before and after treatment; there were 3 biological replicates, totaling 24 samples. Samples were sequenced on the Illumina HiSeq platform (Meiji, Shanghai, China). The differentially expressed genes (DEGs) between the control group and the salt, alkali and salt–alkali mixed stress groups were identified using the DESeq2 package. The thresholds for classification as a DEG were |log_2_FC| ≥ 1 and adjusted *p* < 0.05.

### 2.7 Yeast one-hybrid assay

The interaction between MtGLK1 and potentially interacting genes was evaluated using the yeast one-hybrid (Y1H) system. The motif fragment of interacting genes was amplified and cloned into the pAbAi vector. The bait vector was digested in BstB I and transformed into Y1HGold yeast cells. The full-length *MtGLK1* coding sequence was cloned into the pGADT7 vector, generating the pGADT7-MtGLK1 prey vector. The pGADT7-MtGLK1 and bait vectors were co-transformed into Y1HGold yeast cells. For yeast transformation, 100 µL of Y1HGold competent cells thawed on ice were mixed sequentially with 2 µg plasmid DNA, 10 µL Carrier DNA (denatured at 95 °C for 5 min, snap-chilled on ice, and the step repeated once), and 500 µL PEG/LiAc solution. After mixing, Cells were incubated at 30 °C for 30 min followed by 42 °C for 15 min. Cells were pelleted by centrifugation at 6000 rpm for 40 s, and the supernatant was discarded. The cell pellet was washed with 400 µL ddH_2_O, centrifuged for 30 s, and the supernatant was removed. The yeast cells were resuspended in 50 µL ddH_2_O and cultured on SD/-Ura medium at 28 °C for 72 h. A positive interaction was confirmed on SD-Leu medium containing Aureobasidin A (AbA). The primers used for cloning are listed in **Supplementary Table 1**.

## 3 Results

### 3.1 *MsGLK1* responds to alkali and salt–alkali mixed stresses in alfalfa

Our previous transcriptomes and proteomes analysis of alfalfa under alkali and salt–alkali mixed stresses, identified *MsGLK1* as a potential gene that responded to alkali and salt–alkali mixed stresses (**Supplementary Figure 1**). *MsGLK1* coding sequence (1272 bp in length and encoded 423 amino acids) was isolated via RT-PCR. Sequence analyses revealed that the MsGLK1 protein is overall classified as a G2-like transcription factor family (positions 26–421 aa), and the region of amino acids 141–163 carries a nuclear localization signal (NLS), followed by the amino acid region 163–226 which contains a Myb_DNA binding domain. (**Supplementary Figure 2A**). To further clarify the sequence conservation of MsGLK1 with other GLK proteins, a multiple sequence alignment was conducted on GLK protein sequences from tomato (*Solanum lycopersicum*), rice (*Oryza sativa*), corn (*Zea mays*), *Arabidopsis thaliana*, *M. truncatula*, and alfalfa. The alignment results revealed that all the participating GLK proteins exhibited high conservation in Myb_DNA binding domain, PRD domain and C-terminal GCT-box (**Supplementary Figure 2B**). Based on the above multiple sequence alignment results, a phylogenetic tree was constructed. In the phylogenetic tree, MsGLK1 clustered most closely with its ortholog MtGLK1 from *M. truncatula.* (**Supplementary Figure 2C**).

RT-qPCR analysis showed that under normal conditions, *MsGLK1* could be detected in all tissues, and the expression level in leaves was the highest, the expression levels in roots, stems, and flowers were lower (**Figure 1A**). To investigate the transcriptional response of *MsGLK1* to alkali and salt–alkali mixed stresses, the relative expression levels of *MsGLK1* at four time points (0 h, 24 h, 48 h, and 72 h) were analyzed. Under alkali and salt–alkali mixed stresses, *MsGLK1* expression decreased with the increase in treatment time (**Figure 1B**). The results suggest that *MsGLK1* might be involved in the adaptive regulation of alfalfa alkali under and salt–alkali mixed stresses. Furthermore, to determine the subcellular localization of the MsGLK1 protein in plant cells, a p35S:*MsGLK1*-eYFP fusion vector was introduced into tobacco leaf epidermal cells by *Agrobacterium*-mediated transient transformation. The results confirmed that MsGLK1 was localized in the nucleus (**Figure 1C**).

**Figure 1:**
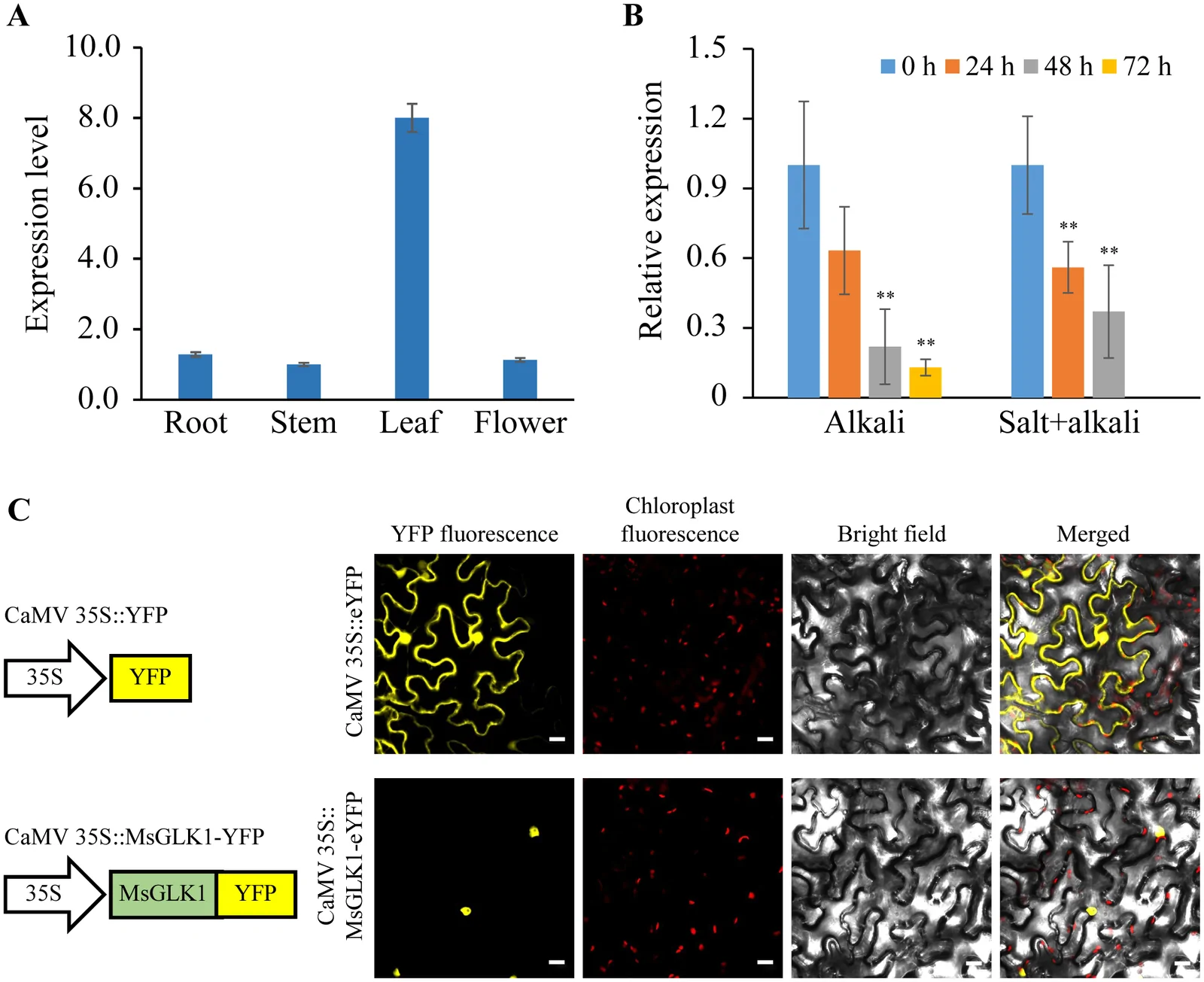
Expression pattern and subcellular localization of *MsGLK1*. **(A)** Expression level of *MsGLK1* gene in alfalfa organs. Error bars indicate SD (n = 3). **(B)** *MsGLK1* expression pattern under alkali and salt-alkali mixed treatment for 0, 24, 48 and 72 h. Error bars indicate SD (n = 3). ** *P* < 0.01. **(C)** Subcellular localization of MsGLK1. Images from left to right represent the yellow fluorescence, chloroplast fluorescence, bright field and merged. Bars = 50 μm.

### 3.2 *Arabidopsis atglk* mutant is sensitive to alkali and salt–alkali mixed stress

To quickly characterize the conserved role of *GLK1* in alkali and salt–alkali mixed stress tolerance, three *Arabidopsis* mutant lines (*atglk1*, *atglk2* and *atglk1/2*) were evaluated. On ½ MS medium, no significant differences in the germination rate were observed between mutants and WT (**Figures 2A, D**). However, under alkali stress, the germination rate of the *atglk1/2* mutant was significantly lower than that of WT from days 2 to 5, with an extremely significant difference on day 3. By day 7, 36.67% of the leaves of the *atglk1/2* mutant displayed purple (**Figures 2B, E**). Similarly, under salt–alkali mixed stress, the germination rate of the *atglk1/2* mutant was significantly lower than that of WT from days 2 to 5, with an extremely significant difference on day 3. After 7 days of salt–alkali mixed stress treatment, the mortality rates of WT, *atglk1*, *atglk2* and *atglk1/2* were 8.33%, 42.31%, 24.00% and 65.38%, respectively (**Figures 2C, F**). The fresh weight and root length of the *atglk1/2* mutant were significantly reduced relative to WT under alkali and salt–alkali mixed stresses (**Figures 2G–K**).

**Figure 2:**
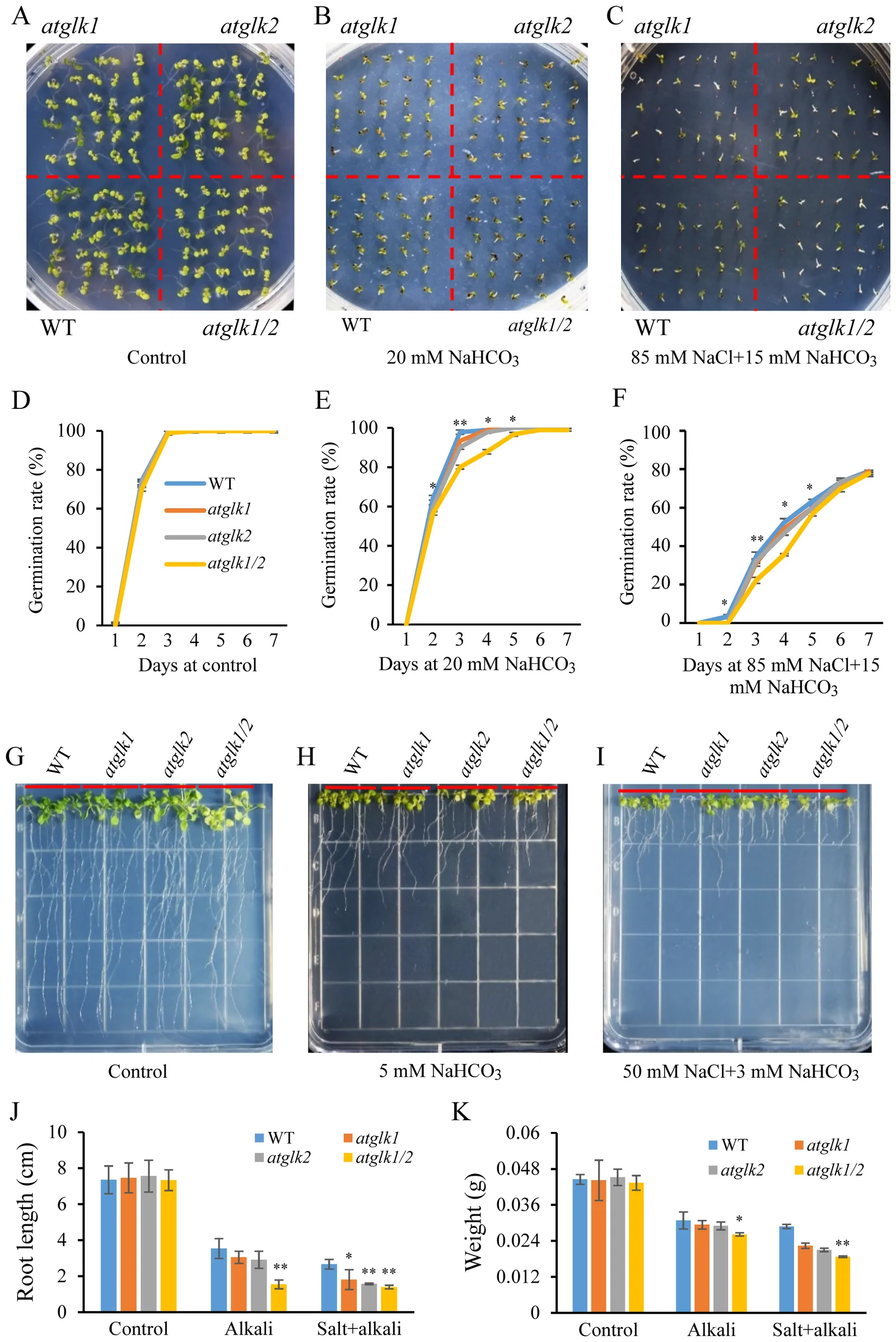
Evaluation of the alkali and salt -alkali mixed tolerance of *Arabidopsis atglk* mutants on culture media. **(A–C)** Germination phenotype of WT, *atglk1*, *atglk2* and *atglk1/2* mutant under control **(A)**, 20 mM NaHCO_3_ **(B)** and 85 mM NaCl + 15 mM NaHCO_3_ **(C)**. **(D–F)** Germination rate under the corresponding stress of **(A–C)**. **(G–I)** Root growth under control **(G)**, 5 mM NaHCO_3_ **(H)** and 5 mM NaCl + 3 mM NaHCO_3_ **(I)**. **(J, K)** Analysis of Root length **(J)** and fresh weight **(K)** of seedlings shown in **(G–I)**. Germination rates were calculated based on 30 seeds for each genotype under each treatment. Error bars indicate SD (n = 3). * *P* < 0.05; ** *P* < 0.01.

Soil-grown *Arabidopsis* WT, *atglk1*, *atglk2* and *atglk1/2* mutant plants were subjected to alkali and salt–alkali mixed stresses. After alkali treatment for 21 days, *atglk1*, *atglk2* mutants were severely damaged, but *atglk1/2* mutant exhibited comparable growth phenotype to that of WT; after salt–alkali mixed treatment for 21 days, *atglk1*, *atglk2* and *atglk1/2* mutants exhibited severe damage (**Figures 3A–C**). When alkali and salt–alkali mixed treatments lasted for 27 days, *atglk1*, *atglk2* and *atglk1/2* mutants exhibited more severe damage than that observed at 21 days, and *atglk1/2* mutant showed purple color under alkali treatment (**Figures 3A, B**). After treatments lasted for 27 days, the survival rates of *atglk1, atglk2 and atglk1/2* mutants were significant lower than WT under alkali and salt–alkali mixed treatments (**Figure 3C**). To characterize the salt stress response of *atglk* mutants, salt stress phenotyping assays were performed. Under salt stress, the *atglk1/2* mutant exhibited significantly reduced germination rate, root length and survival rate relative to WT, whereas no obvious difference in fresh weight was observed between the mutant and WT (**Supplementary Figure 3**).

**Figure 3:**
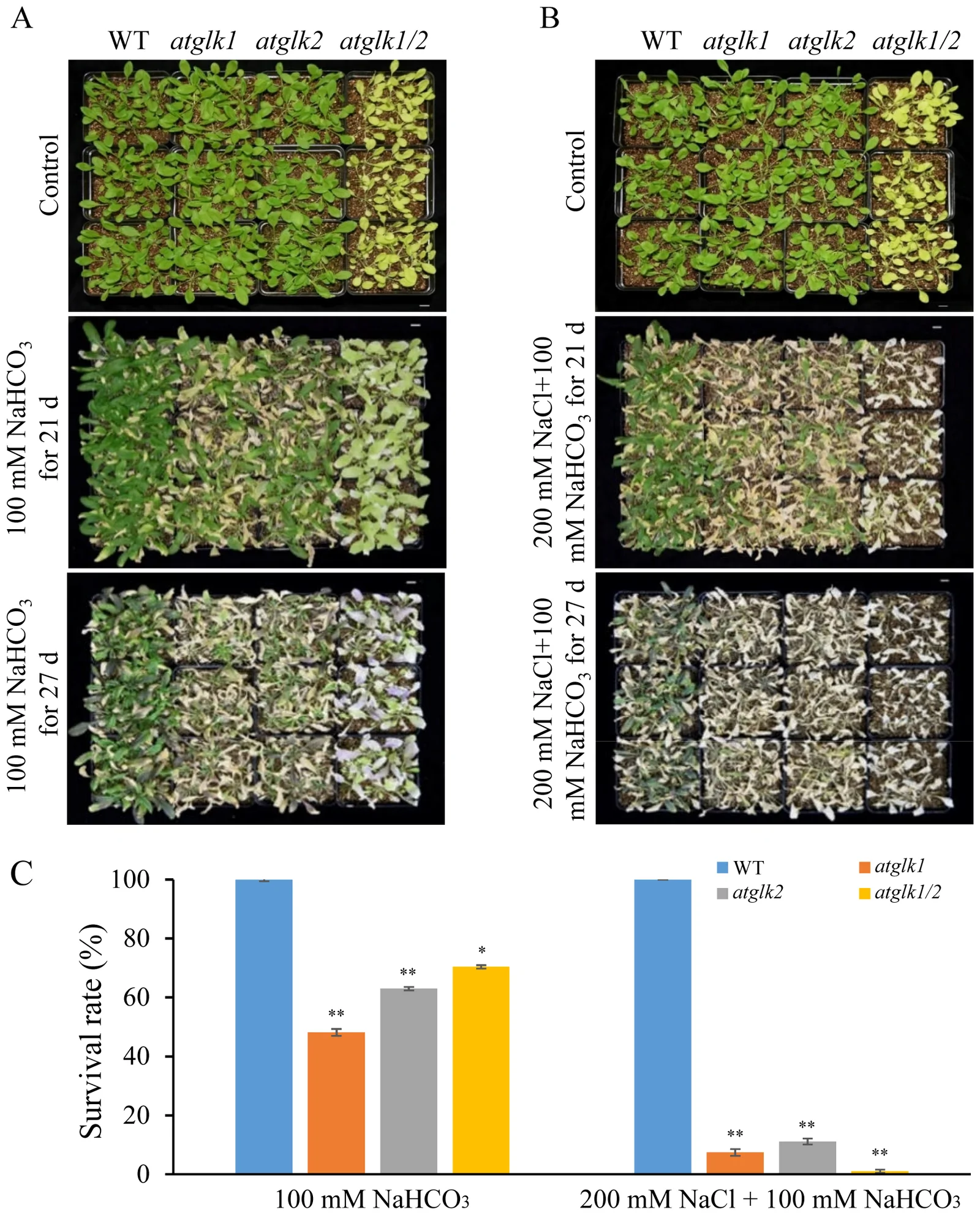
Evaluation of the alkali and salt -alkali mixed tolerance of *Arabidopsis atglk* mutant plants. **(A, B)** Phenotype of soil-grown WT and *atglk* mutant plants subjected to alkali treatment **(A)** and salt -alkali mixed treatment **(B)**. **(C)** Survival rate after 27 days of alkali and salt-alkali mixed stresses. Error bars indicate SD (n = 3). * *P* < 0.05; ** *P* < 0.01.

### 3.3 Overexpression of *MsGLK1* in *Arabidopsis* confers tolerance to alkali and salt–alkali mixed stresses

The p35S:*MsGLK1*-eYFP vector was transformed into *Arabidopsis*, transgenic lines were identified by PCR (**Supplementary Figure 4A**), and the transcriptional expression of *MsGLK1* was confirmed by RT-qPCR (**Supplementary Figure 4B**). Three overexpression lines (OE5, OE12 and OE17) with elevated expression levels were selected for subsequent experiments. On ½ MS medium, no significant differences were observed in root length and fresh weight between OE lines and WT (**Supplementary Figures 4C–F**). Under alkali and salt–alkali mixed stress, the root elongation of WT seedlings was severely inhibited. In contrast, the OE lines developed significantly longer roots and higher fresh weight than WT (**Figures 4A–E**). In soil experiment, 3-week-old soil-grown *Arabidopsis* OE and WT plants were subjected to stresses, no obvious difference was observed (**Supplementary Figures 4G–I**). The oxidative stress indicators and chlorophyll content were measured after alkali and salt–alkali mixed treatments for 6 days. Under alkali and salt–alkali mixed stress, the OE lines exhibited a significant reduction in MDA content, coupled with a significant increase in CAT content and SOD activity. Under alkali and salt–alkali mixed stresses, the OE lines maintained significantly higher chlorophyll content than the WT. Physiological data showed that overexpression of *MsGLK1* in *Arabidopsis* elicits a protective response to alkali and salt–alkali mixed stresses (**Figure 5**). To investigate the effect of *MsGLK1* overexpression on salt tolerance in *Arabidopsis*, we performed salt stress assays. Under salt treatment, no significant differences in root length, fresh weight, survival rate, and the physiological parameters were observed between the OE lines and WT plants (**Supplementary Figure 4J**). These results demonstrated that overexpression of *MsGLK1* in *Arabidopsis* conferred tolerance to alkali and salt–alkali mixed stresses, but did not alter response to salt stress.

**Figure 4:**
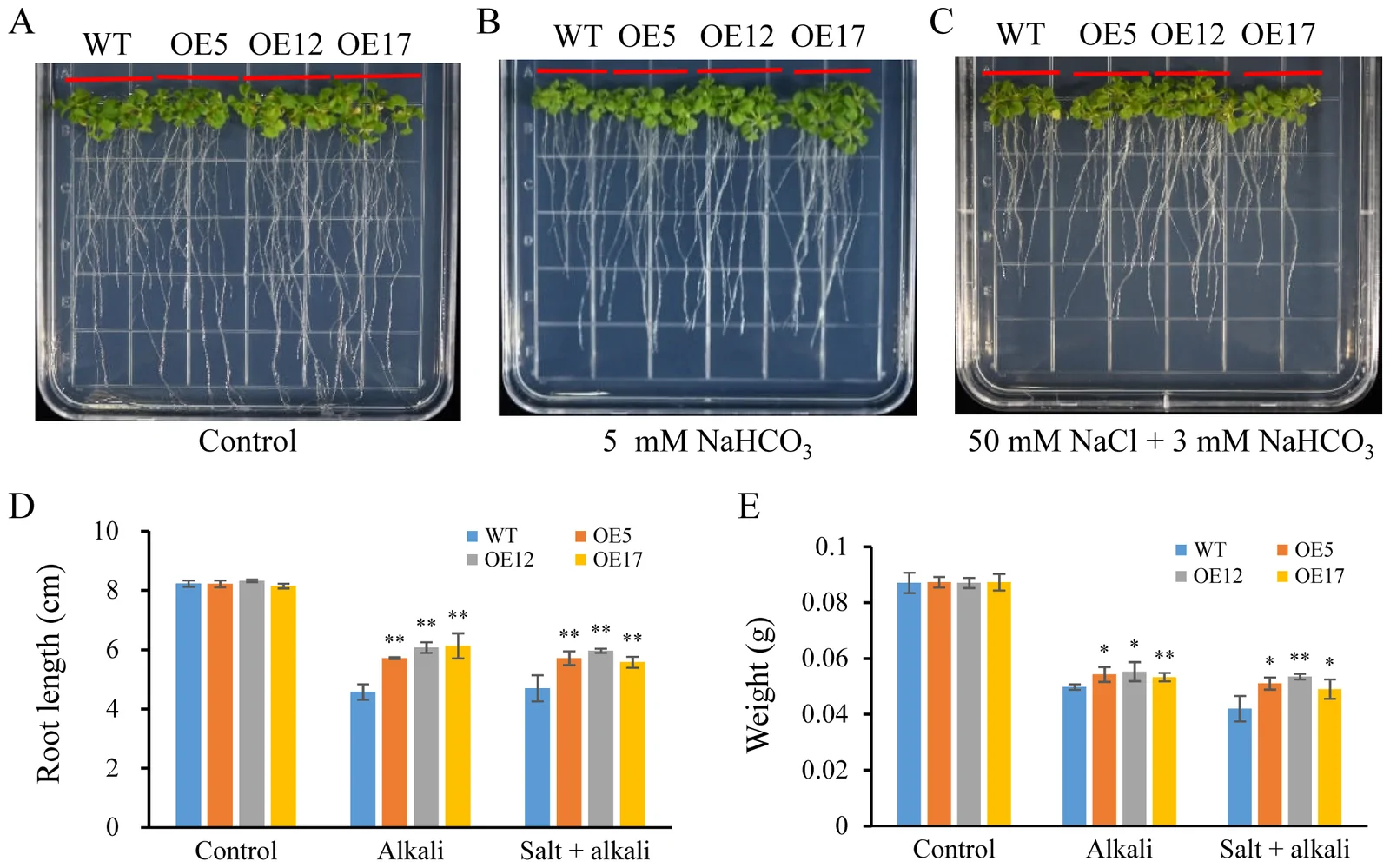
Overexpression of *MsGLK1* in *Arabidopsis* enhanced alkali and salt -alkali mixed tolerance on culture media. **(A–C)** Root growth under control **(A)**, 5 mM NaHCO_3_ **(B)** and 5 mM NaCl + 3 mM NaHCO_3_ **(C)**. **(D, E)** Analysis of Root length **(D)** and fresh weight **(E)** of seedlings. Error bars indicate SD (n = 3). * *P* < 0.05; ** *P* < 0.01.

**Figure 5:**
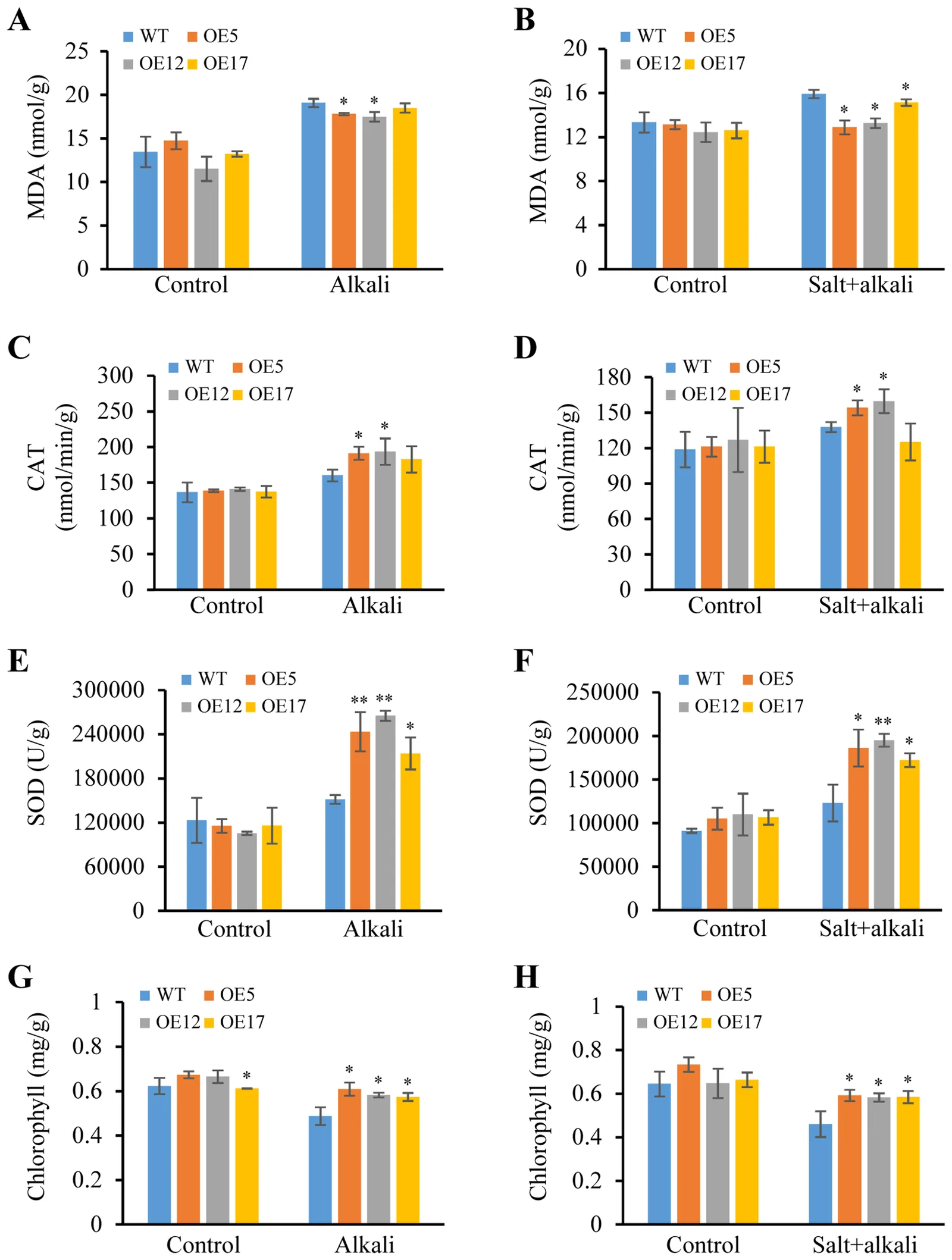
*MsGLK1* modulates stress physiology in transgenic *Arabidopsis*. **(A, C, E, G)** Level of malondialdehyde (MDA) **(A)**, activity of catalase (CAT) **(C)**, superoxide dismutase (SOD) **(E)** and chlorophyll content **(G)** in WT and *MsGLK1*-overexpression (OE) *Arabidopsis* plants under alakli stress. **(B, D, F, H)** Level of MDA **(B)**, activity of CAT **(D)**, activity of SOD **(F)** and chlorophyll content **(H)** in WT and *MsGLK1*-overexpression (OE) *Arabidopsis* under salt -alkali mixed stress. Error bars indicate SD (n = 3). * *P* < 0.05; ** *P* < 0.01.

### 3.4 The *mtglk1* mutant in *M. truncatula* is sensitive to alkali and salt–alkali mixed stresses

To characterize *GLK1* function in *M. truncatula*, we identified a *mtglk1* mutant (NF20408) line. PCR analysis confirmed that the *Tnt1* retrotransposon was inserted in the first exon, and RT-PCR analysis demonstrated the absence of full-length *MtGLK1* transcripts in homozygous mutant plants (**Figures 6A–C**). To determine the tolerance of the *atglk1* mutant and WT plants to alkali and salt–alkali mixed treatments, plants were grown under normal conditions for 30 days before being subjected to alkali (100 mM NaHCO_3_) and salt–alkali mixed (200 mM NaCl + 100 mM NaHCO_3_) treatments. Before treatment, no obvious morphological differences were observed between *mtglk1* mutant and WT plants. After 14 days of alkali and salt–alkali mixed stresses, the *mtglk1* plants exhibited complete mortality (**Figures 6D, E**). The results revealed that the loss of function of *MtGLK1* leads to an obvious decrease in the tolerance of the *mtglk1* mutant to alkali and salt–alkali mixed stresses.

**Figure 6:**
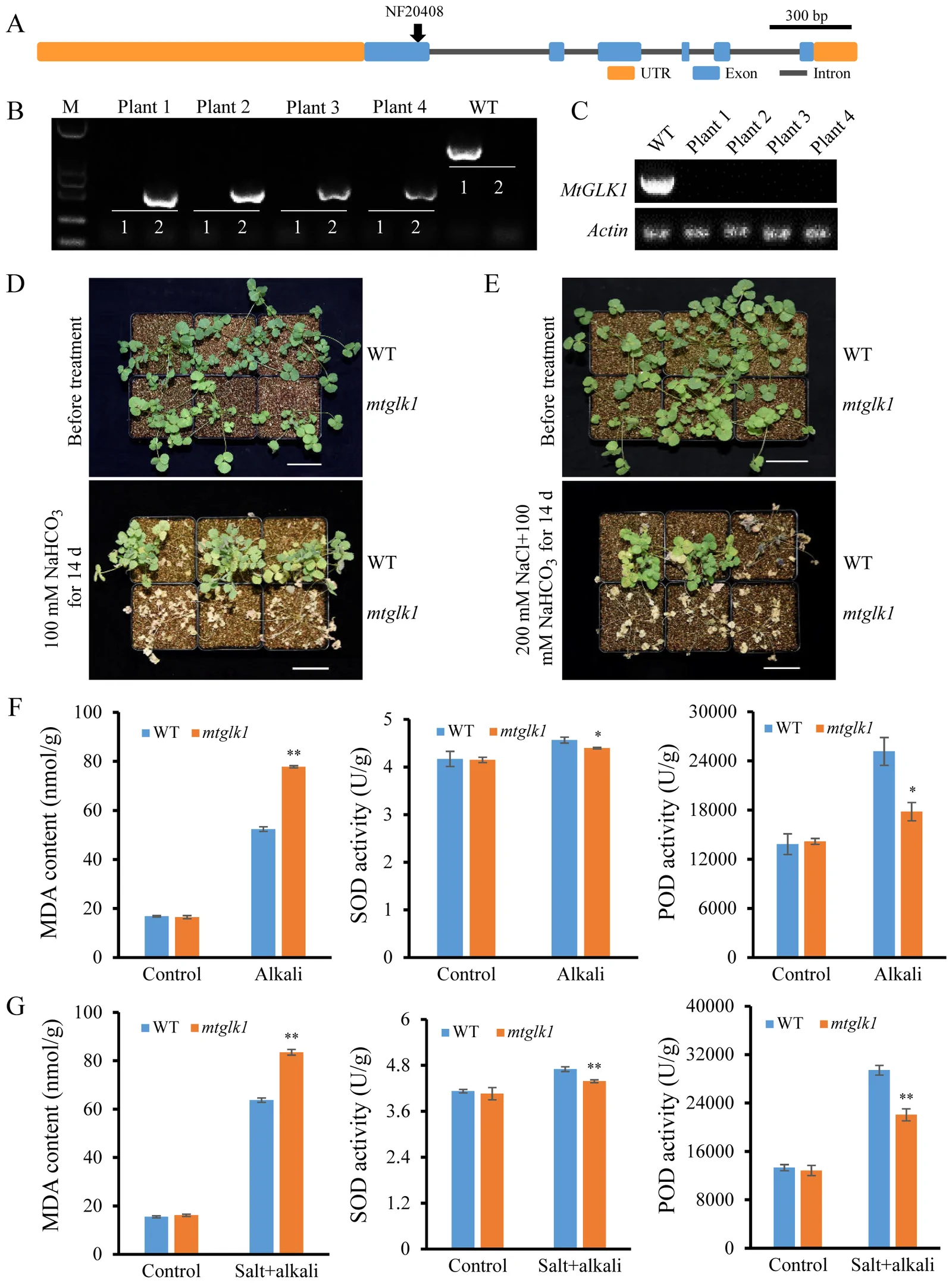
Evaluation of alkali and salt -alkali tolerance of *mtglk1* mutant plants in *M. truncatula*. **(A)** Diagram of the *Tnt1* insertion sites of *mtglk1* mutant. **(B)** PCR verification of the homozygous *mtglk1* mutant. **(C)** RT-qPCR verification of the homozygous *mtglk1* mutant. **(D, E)** Phenotype of WT and *mtglk1* mutant plants before (upper panels) and after treatment (lower panels) with 100 mM NaHCO_3_ **(D)** and 150 mM NaCl + 100 mM NaHCO_3_ **(E)**. Scale bar = 5 cm. **(F, G)** Physiological indices of oxidative stress in leaves of WT and *mtglk1* mutant after 5 days of alkali stress **(F)** and salt-alkali mixed stress **(G)**. Error bars indicate SD (n = 3). * *P* < 0.05; ** *P* < 0.01.

The physiological parameters were measured 3 days after treatments. Under alkali and salt–alkali mixed stresses, *mtglk1* mutant exhibited a significant elevated MDA level, accompanied by markedly reduced activities of SOD and POD (**Figures 6F, G**). To characterize the salt stress response of *mtglk1* mutants, salt treatment was conducted. No significant phenotypic differences were observed between *mtglk1* mutant and WT plants before and after salt treatment, and no differences in MDA content, SOD activity, or POD activity were detected between the *mtglk1* mutant and WT plants (**Supplementary Figure 6**). These data demonstrated that the loss of *MtGLK1* impairs antioxidant capacity, resulting in enhanced membrane oxidation damage and sensitivity to alkali and salt–alkali mixed stresses.

### 3.5 Gene expression analysis identified genes regulated by *GLK1* under salt and alkali stresses

To explore the regulatory network controlled by *MtGLK1*, RNA‐seq was performed on *mtglk1* and WT under different conditions. Two independent comparison strategies were adopted for transcriptome analysis. The comparisons evaluated transcriptional changes induced by stress within the same genotype, including to SWT vs CKWT, AWT vs CKWT, SAWT vs CKWT for WT plants, and S*mtglk1* vs CK*mtglk1*, S*mtglk1* vs CK*mtglk1*, SA*mtglk1* vs CK *mtglk1* for *mtglk1* mutant plants (**Figure 7A**). The comparisons characterized the transcriptional differences between *mtglk1* and WT under identical growth conditions, namely CK*mtglk1* vs CKWT (control), S*mtglk1* vs SWT (salt), A*mtglk1* vs AWT (alkali), SA*mtglk1* vs SAWT (salt–alkali mixed). Under control, salt, alkali and salt–alkali mixed conditions, 408 (242 upregulated and 166 downregulated), 39 (29 upregulated and 10 downregulated), 1968 (377 upregulated and 1591 downregulated) and 3632 (2015 upregulated and 1617 downregulated) DEGs, respectively, were identified between WT and *mtglk1* (**Figure 7A**). The Venn diagram showed that 5, 1092 and 999 DEGs were uniquely regulated by salt, alkali and salt–alkali mixed stresses in *mtglk1* mutant, respectively (**Figure 7B**).

**Figure 7:**
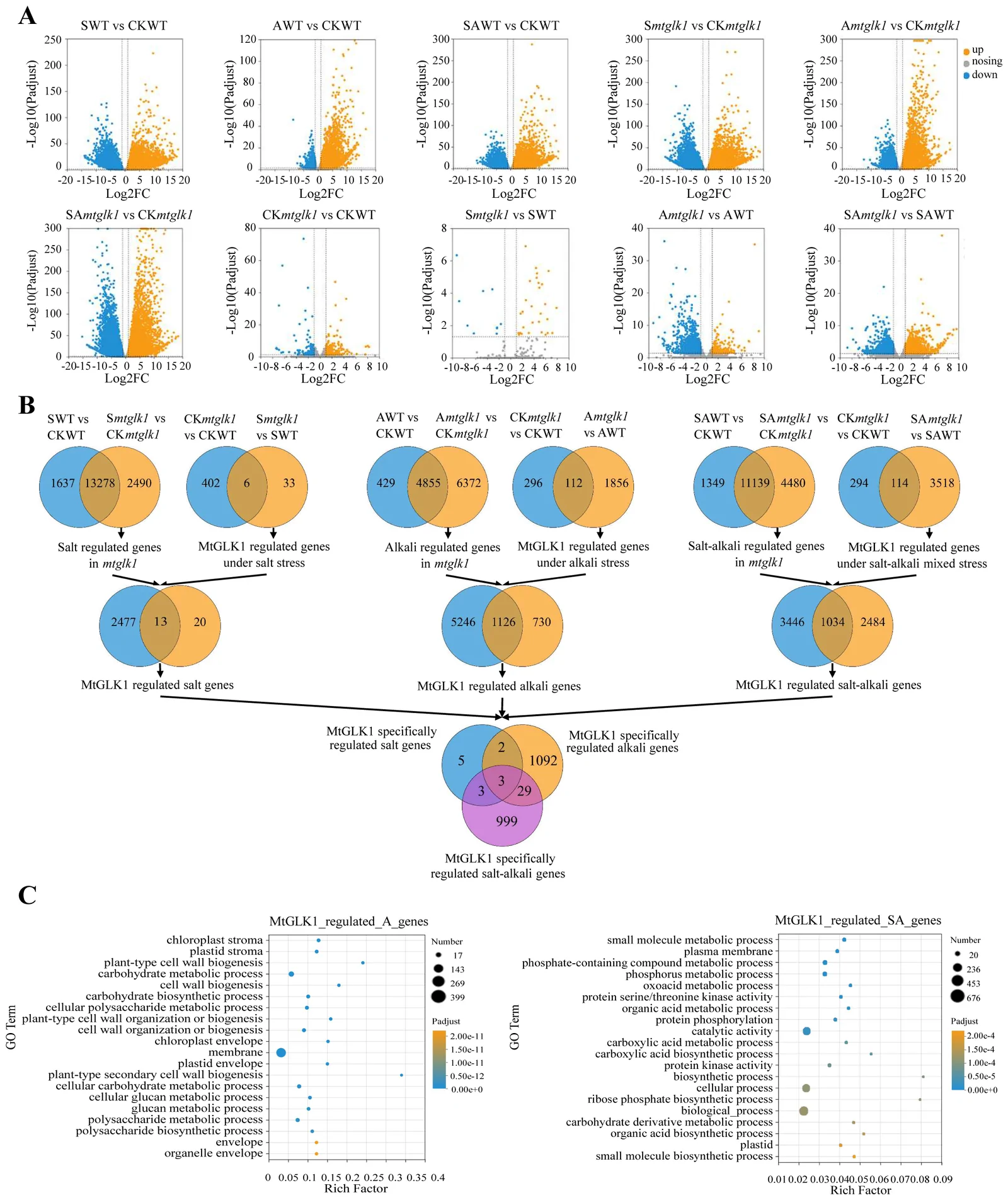
Transcriptome analysis of *mtglk1* mutant under salt, alkali and salt-alkali mixed stress. **(A)** Volcano plots represent the fold -change of differentially expressed genes (DEGs) in comparison of SWT versus CKWT, AWT versus CKWT, SAWT versus CKWT, S*mtglk1* versus CK*mtglk1*, A*mtglk1* versus CK*mtglk1*, SA*mtglk1* versus CK*mtglk1*, CK*mtglk1* versus CKWT, S*mtglk1* versus SWT, A*mtglk1* versus AWT, SA*mtglk1* versus SAWT (*P* < 0.05, absolute fold change ≥ 2.0). **(B)** Venn diagrams represent DEGs regulated by salt, alkali, and salt -alkali mixed stresses and *MtGLK1*. **(C)** GO analysis shows top 20 enriched terms of DEGs under alkali and salt -alkali mixed stress. CK: control condition. S: 300 mM NaCl for 3 days. A: 100 mM NaHCO_3_ for 3 days. SA: 200 mM NaCl + 100 mM NaHCO_3_ for 3 days. *mtglk1*: *Tnt1* insertion mutant; WT: wild type.

Gene Ontology (GO) analysis showed that DEGs specifically regulated by alkali stress were enriched in the processes of chloroplast stroma (GO: 0009570), cell wall organization or biogenesis (GO: 0071554), and membrane (GO: 0016020). Most DEGs regulated by salt–alkali mixed stress were enriched in the processes of plasma membrane (GO: 0005886), phosphorus metabolic process (GO: 0006793), protein phosphorylation (GO: 0006468) and protein kinase activity (GO: 0004672) (**Figure 7C**). Under alkali and salt–alkali mixed stress, the enriched GO terms for DEGs were divergent.

A subset of DEGs encoded TFs was analyzed. 179 and 90 TFs were identified among the DEGs that responded exclusively to alkali and salt–alkali mixed stresses, respectively, in *mtglk1* mutant. We applied a screening strategy to identify TFs potentially under the direct regulation of *MtGLK1*, focusing on those with comparable expression in WT and *mtglk1* mutant under control but showing significant differential expression under alkali, salt–alkali mixed stresses. This approach identified 17 and 16 genes specifically associated with alkali and salt–alkali mixed stresses, respectively (**Figures 8A, B**). These TFs mainly belong to the bHLH, MYB, NAC, AP2 and MADS families (**Supplementary Table 2**). The expression level of selected genes were shown in **Figures 8C, D**. Subsequently, promoter analysis of the seven most highly expressed genes from the alkali stress-specific genes revealed that three genes (Medtr1g023260, Medtr0011s0260 and Medtr1g053800) contain the *GLK1*-binding motif in their promoter regions (**Supplementary Figure 7A**).

**Figure 8:**
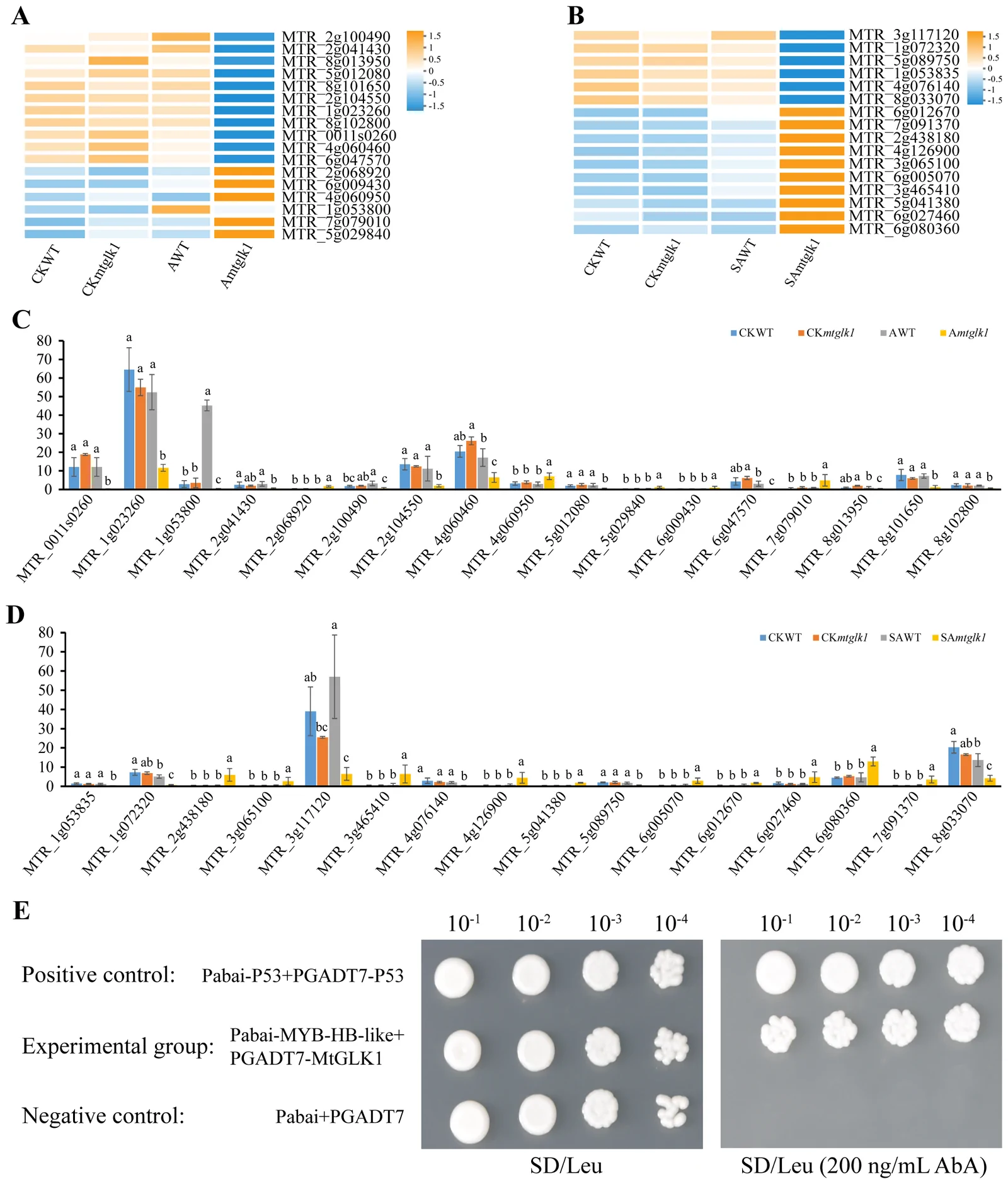
Validation of *MYB-HB-like* as a direct target of MtGLK1. **(A, B)** Heatmap showing expression profiles of transcription factor (TF) genes identified as co-regulated DEGs by both *mtglk1* and alkali stress **(A)** or salt -alkali mixed stress **(B)**. **(C, D)** Expression level of selected TF genes from **(A, B)**. Different lowercase letters indicate significant differences among groups. Error bars indicate SD (n = 3). **(E)** Yeast one-hybrid assay confirming that MtGLK1 binds to the promoter motif of the *MYB-HB-like* gene.

### 3.6 GLK1 interacts with the *MYB-HB-like* promoter in Y1H assays

To confirm the target genes interacting with *MtGLK1*, a prey vector was constructed by cloning CDS sequence of *MtGLK1* into the pGADT7 plasmid and three bait vectors were generated by cloning the promoter motif sequences of the three candidate genes into the pABAi vector (**Supplementary Figure 7B**). The bait vectors were transformed into yeast cell, auto-activation assays were performed, which determined the optimal AbA concentrations for suppression to be 200 ng/mL for *MYB*-*HB*-*like* (Medtr1g053800) gene and 300 ng/mL for *BBX21* (Medtr1g023260) gene and *bHLH25* (Medtr0011s0260) gene (**Supplementary Figure 8A**). Y1H assays were conducted on SD/-Leu medium containing the corresponding AbA concentrations. Yeast cells co-transformed with the pabai-*MYB*-*HB*-*like* bait vector and the pGADT7-MtGLK1 prey vector grew on SD/-Leu medium containing 200 ng/mL AbA (**Figure 8E**), but no interaction was observed with the *BBX21* and *bHLH25* promoters (**Supplementary Figure 8B**). This result revealed that MtGLK1 specifically binds to the GATTCT motif within the *MYB*-*HB*-*like* promoter.

To verify whether the expression of *MtMYB-HB-like* is regulated by MtGLK1 under alkali stress, we performed qRT-PCR to monitor its expression levels over a time-course of alkali treatment (0, 6, 12, 24, and 48 h) in *mtglk1* mutant. Under alkali stress, expression levels of *MtMYB-HB-like* was significantly repressed at early time points (6, 12, and 24 h), followed by partial recovery at 48 h. We also profiled its expression level under salt stress. In contrast to the alkali response, the expression of *MtMYB-HB-like* showed no significant alteration across all time points.

## 4 Discussion

### 4.1 The *MsGLK1* gene from alfalfa

*Golden2-like 1* (*GLK1*), a conserved TF, functions in the regulation of chloroplast development, chlorophyll synthesis, as well as abiotic stress tolerance in green plants (Fitter et al., 2002; Li et al., 2024; Zheng et al., 2024). A typical GLK protein has always retained two highly conserved structure: one is the DBD domain shared with the GARP family, which indicates the common origin of the GLK protein and this family’s ancestor; the other is the C-terminal GCT domain specific to the GLK protein, whose high conservation in different species reflects the strong selective pressure on this domain during the functional specialization of the GLK protein (Alam et al., 2022; Hu et al., 2023). In this study, the *MsGLK1* gene was isolated and characterized from alfalfa, with its coding region being 1272 bp and encoding 423 amino acids. The MYB-DNA binding domain, GCT-box, and PRD domain of MsGLK1 all showed extremely high sequence consistency in different species, indicating the high conservation of the GLK transcription factor family in plants. The presence of the NLS indicates that MsGLK1 can enter the cell nucleus through active transport to perform transcriptional regulatory functions, which is consistent with the localization characteristics of GLK proteins as nuclear transcription factors (Zheng et al., 2024). Consistent with previous studies, sequence analysis showed highly conserved MYB-DNA binding domain and C-terminal GCT-box domains. Evolutionary analysis showed that *MsGLK1* was closely related to *MtGLK1*.

### 4.2 *GLK1* gene regulates the tolerance to alkali and salt–alkali mixed stresses

Previous studies have shown that *GLK1* has a positive effect on tolerance to drought and cold stress (Liu et al., 2018; Ahmad et al., 2019; Liu et al., 2020, 2021). *AhGLK1* was involved in recovery growth after drought, and overexpression of *AhGLK1* improved drought tolerance of transgenic *Arabidopsis* (Liu et al., 2018, 2020). The *atglk1/2* is hypersensitive to abscisic acid in *Arabidopsis*, and *GLK1* or *GLK2* overexpression enhanced abscisic acid tolerance (Ahmad et al., 2019; Lee et al., 2023). In cotton, after reducing *GhGLK1* expression via virus-induced gene silencing, the drought and cold tolerance significantly decreased, while overexpression of *GhGLK1* in *Arabidopsis* enhanced its drought and cold tolerance (Liu et al., 2021). However, the role of *GLK1* in salt and alkali tolerance has not been reported previously.

In this study, the expression level of *MsGLK1* was affected by salt, alkali and salt–alkali mixed stresses, indicating that it is involved in regulating salt and alkali tolerance. It is notable that salt stress and alkali stress have significant differences in essence: salt stress mainly causes ionic toxicity and osmotic stress, while alkali stress adds additional damage on salt stress, including disrupting ion balance, affecting the absorption and utilization of mineral elements, and inhibiting the activity of various enzymes (Zhang et al., 2024). Therefore, alkali stress often causes more severe damage to plants. This background helps to understand the molecular basis of the functional differences of *GLK1* under different stress conditions in this study.

In *Arabidopsis*, the *atglk1/2* mutant was sensitive to salt, alkali and salt–alkali mixed stresses, while the *MsGLK1* overexpression lines were more tolerant to alkali and salt–alkali mixed stresses, but no improvement in salt tolerance. This result indicates that the function of *MsGLK1* does not respond equally to all ion stress, and it has a more specific regulatory role in alkali stress. Consistent with this finding, the *mtglk1* mutant of *M. truncatula* showed no obvious response to salt stress, but it was very sensitive to alkali and salt–alkali mixed stresses. After 14 days of treatment, it exhibited an extreme phenotype of complete death, further supporting the function of *GLK1* in the response to alkali stress.

From a physiological perspective, under the conditions of alkali and salt-alkali mixed stress, the increased MDA content and reduced SOD and POD activities in the *mtglk1* mutant indicate that the absence of *MtGLK1* leads to impaired antioxidant capacity in the plants and aggravated membrane lipid peroxidation damage. Correspondingly, the overexpressing *MsGLK1 Arabidopsis thaliana* lines exhibited decreased MDA content, increased CAT content and SOD activity, and maintained relatively high chlorophyll contents under alkali and salt-alkali mixed stress.

These physiological changes correlate with stress performance. It is plausible that *MsGLK1* contributes to stress adaptation, possibly through modulation of antioxidant defense and photosynthetic integrity. The maintenance of chlorophyll content is particularly important, which is highly consistent with the classical function of *GLK1* as a transcriptional activator of photosynthesis-related genes, indicating that *MsGLK1* may alleviate oxidative damage caused by alkali stress by maintaining the integrity of the photosynthetic function. Given the great importance of knockout of endogenous *MsGLK1* in alfalfa would be valuable for fully dissecting its biological function. CRISPR/Cas9-editing vectors have been constructed, and the *Agrobacterium-mediated* transformation for *MsGLK1* knockout in alfalfa has been completed. Nevertheless, owing to the long regeneration cycle of autotetraploid alfalfa, resistant callus screening is still in progress, and regenerated knockout plants suitable for phenotypic characterization have not been acquired. Generating *MsGLK1* knockout lines will further elucidate the biological roles of *MsGLK1* under salt-alkali stress. The transcriptome analysis results further validated the above conclusion. Compared with salt stress (only 5 specific DEGs were identified), under alkali stress and salt-alkali mixed stress, 1092 and 999 specific differentially expressed genes were identified in the *mtglk1* mutant. The significant difference in the number of DEGs directly reveals the regulatory role of *GLK1* in the alkali stress response, and also explains why the *glk1* mutant shows more significant sensitivity to alkali stress rather than salt stress. Divergent GO enrichment profiles further revealed distinct stress response strategies underlying alkali and salt-alkali mixed stress. Alkali stress mainly causes high-pH and bicarbonate toxicity; accordingly, alkali-responsive DEGs were enriched in cell wall biogenesis, carbohydrate and polysaccharide metabolism, and chloroplast/plastid membrane homeostasis pathways, which contribute to maintaining cell wall stability and plastid integrity under high-pH stress. In comparison, salt-alkali mixed stress combines high-pH and bicarbonate damage with additional Na^+^ toxicity. Excess Na^+^ activates ion-sensing kinase cascades, leading to extensive protein phosphorylation and phosphorus metabolic reprogramming. Moreover, plants mobilize organic acid metabolism to accumulate organic anions for intracellular pH buffering, which accounts for the significant enrichment of kinase activity, protein phosphorylation, and organic acid metabolism pathways under mixed stress. The distinct ionic and physicochemical properties of the two stress types trigger different upstream signaling events, thereby driving divergent GLK1-dependent transcriptional networks. Based on the comprehensive phenotypic, physiological and transcriptomic data, this study clearly confirms that *GLK1* is a factor regulating the tolerance of plants to alkali and salt-alkali mixed stress.

In alfalfa, the MsCDPK29-MsbZIP14 module enhances saline-alkali tolerance mainly by promoting ABA biosynthesis (Ren et al, 2026), while *MsNF-YC2* improves alkali resistance by regulating hormone signaling and photosynthetic pathways (Yu et al., 2022). The plasma membrane-localized MsRCI2A/B/C proteins mainly maintain ion and membrane homeostasis, with stronger effects on salt stress than weak alkali stress and functional redundancy among family members (Li et al., 2021). In contrast, *GLK1* exhibits a more specific and dominant regulatory role in alkali stress responses. The *mtglk1* mutant displayed severe damage under alkali and salt–alkali mixed conditions, indicating that GLK1 is an indispensable core regulator for alkali adaptation. Different from the ABA-dependent pathways of previously reported alfalfa genes, *MsGLK1* confers alkali tolerance mainly by maintaining photosynthetic integrity and antioxidant homeostasis. Moreover, *GLK1* specifically responds to alkali stress rather than salt stress.

### 4.3 MtGLK1 directly binds to the *MtMYB-HB-like* gene

TFs play crucial roles in enhancing plant stress tolerance (Bhat et al., 2024). To elucidate the downstream regulatory mechanism of *MtGLK1*-mediated alkali stress tolerance, this study conducted a systematic analysis of the transcriptome data of the *mtglk1* mutant. By screening transcription factors that showed no significant difference in expression between WT and mutant under normal conditions but exhibited significant differential expression under alkali stress or salt-alkali mixed stress, a total of 17 and 16 candidate TF genes specifically responding to alkali stress or salt-alkali mixed stress were identified, respectively. The selected candidate genes are more likely to represent the direct or indirect regulatory targets of *MtGLK1* under stress conditions.

Previous studies have shown that GLK1 can specifically recognize the sequence CCAATC, CACGTG, GATTCT and GATATC in the promoter region of target genes (Waters et al., 2009; Nagatoshi et al., 2016; Lee et al., 2023; Li et al., 2024). In this study, the promoter analysis of the seven high-expression candidate genes was conducted, and it was found that the promoter regions of *MtMYB-HB-like* contain GLK1-binding core sequence, indicating that *MtMYB-HB-like* may be targets of MtGLK1. The Y1H experimental results confirmed that MtGLK1 directly binds to the promoter of *MtMYB*-*HB*-*like*. The result suggested that GLK1 confer alkali tolerance by regulating the expression of *MtMYB-HB-like*. qRT-PCR analysis showed that *MtMYB-HB-like* was significantly repressed during the early phase of alkali stress and partially recovered at 48 h. These results support a putative regulation between MtGLK1 and *MtMYB-HB-like* specifically under alkali stress. Although Y1H assays and qRT-PCR analysis support the interaction of MtGLK1 and *MtMYB-HB-like*, further EMSA and ChIP are still required to verify this physical interaction under native plant conditions.

MYB TFs constitute one of the largest gene families in plants, and play critical roles in plant growth and development, cell morphogenesis, metabolism, and adaptation to abiotic stresses. As a member of the MYB superfamily, *MYB-HB-like* belongs to the 1R-MYB (MYB-related) subfamily, which is structurally distinct from the well-characterized R2R3-MYB proteins. Several studies have demonstrated that 1R-MYB proteins participate in responses to multiple abiotic stresses. For instance, *MaMYBR30* positively regulates drought tolerance but negatively modulates salt resistance in mulberry (Liu et al., 2024). *SlMYB1L* confers drought resistance by mediating stomatal closure and endogenous ABA, and *FvMYB114* is implicated in salt and cold stress adaptation in transgenic *Arabidopsis* (Li et al., 2023; Liu et al., 2025). Despite these advances, no functional characterization of *MtMYB-HB-like* has been reported to date, and its biological function remains largely unknown. The biological function and its downstream target gene network of *MtMYB-HB-like* gene in alkali stress deserves further research.

## 5 Conclusion

This study reveals the new role of *GLK1* in regulating plant tolerance to alkali and salt–alkali mixed stress. Loss-of-function of *GLK1* in *Arabidopsis* (*atglk1/2*) and *M. truncatula (mtglk1*) shows reduced tolerance to alkali and salt–alkali mixed stresses, while overexpression of *MsGLK1* in *Arabidopsis* exhibits enhanced tolerance to alkali and salt–alkali mixed stresses. RNA-seq analysis demonstrates that mutation of *MtGLK1* gene resulted in differential expression of 1,092 and 999 genes uniquely under alkali and salt–alkali mixed stresses, respectively, but only 5 genes under salt stress. Furthermore, a direct downstream target by showing that *MtGLK1* physically binds to the promoter of the *MtMYB-HB-like* gene via a specific GATTCT motif. This work provides novel insights into the function of the *GLK1* gene.

## Data Availability

The raw RNA-seq reads generated in this study were deposited in the NCBI Sequence Read Archive (SRA) database under BioProject accession PRJNA1525638, with BioSample accessions SAMN63014413–SAMN63014436.

## Supplementary material

Supplementary Figure 1 *MsGLK1* expression pattern in the transcriptome of alfalfa subjected to salt–alkali treatment. CK: ½ Hoagland solution. A: 50 mM NaHCO_3_. 90 S + 10 A: 90 mM NaCl+10 mM NaHCO_3_. 80 S + 20 A: 80 mM NaCl+20 mM NaHCO_3_. 70 S + 30 A: 70 mM NaCl+30 mM NaHCO_3_. 60 S + 40 A: 60 mM NaCl+40 mM NaHCO_3_. The asterisks indicate significant differences between different treatment times under the same treatment. * *P* < 0.05; ** *P* < 0.01. The letters represent significant differences between different treatments under the same treatment time. Error bars indicate SD (n = 3).

Supplementary Figure 2 Identification of *MsGLK1*. **(A)** Graphical representation of the MsGLK conserved domain. Myb -binding domain and Golden-2 like transcription factor are the major domains. **(B)** Multiple sequence alignment of GLK1 and GLK2 proteins. The putative Myb-binding domain folds as an HLH. Green lines indicate predicted α-helix segments conserved in GLK proteins. Purple and blue lines indicate proline rich domain (PRD) and GLK/C-terminal box (GCT-box) region, respectively. **(C)** Evolutionary tree of GLK1 and GLK2 proteins. GLK proteins from *Solanum lycopersicum* (SlGLK1, NP_001266193.1; SlGLK2, NP_001266252.1), *Oryza sativa* (OsGLK1, NP_001389733.1; OsGLK2, NP_001384874.1), *Zea mays* (ZmGLK1, NP_001105018.1; ZmGLK2, NP_001105513.1), *Arabidopsis thaliana* (AtGLK1, NP_565476.1; AtGLK2, and NP_199232.1) and *Medicago truncatula* (MtGLK1, XP_013465151.1).

Supplementary Figure 3 Evaluation of the salt tolerance of *Arabidopsis atglk* mutants. **(A, B)** Germination phenotype of WT and *atglk* mutants under control **(A)** and 150 mM NaCl **(B)**. **(C, D)** Germination rate corresponding stress of **(A, B)**. **(E, F)** Root growth under control **(E)**, 150 mM NaCl **(F)**. **(G, H)** Root length **(G)** and fresh weight **(H)** of seedlings shown in **(E, F)**. **(I, J)** WT and *atglk* mutant seedlings under control and salt stress. **(L)** Survival rate after 27 days of salt stress. Error bars indicate SD (n = 3). * *P* < 0.05; ** *P* < 0.01.

Supplementary Figure 4 Evaluation of the salt tolerance in *MsGLK1* overexpression *Arabidopsis* lines. **(A)** PCR verification of *MsGLK1* gene and the marker gene (Bar) in *MsGLK1* overexpression *Arabidopsis* lines. P: pFGC-MsGLK1 vector plasmid (positive control); WT: wild type *Arabidopsis* (negative control); M: DNA marler. **(B)** Expression level of *MsGLK1* in *MsGLK1* overexpression *Arabidopsis* lines. Error bars indicate SD (n = 3). **(C, D)** Root growth under control **(C)**, and 150 mM NaCl **(D)**. **(E, F)** Analysis of root length **(E)** and fresh weight **(F)** of seedlings under salt stress. (G-1) Phenotype of soil-grown WT and *MsGLK1* overexpression *Arabidopsis* lines subjected to treatment of 300 mM NaCl **(G)**, 100 mM NaHCO_3_ **(H)** and 200 mM NaCl + 100 mM NaHCO_3_ **(I)**. **(J)** Level of MDA, activity of CAT, activity of SOD and chlorophyll content in WT and OE lines under salt stress. Error bars indicate SD (n = 3). * *P* < 0.05.

Supplementary Figure 5 Evaluation of the salt tolerance of *mtglk1* mutant in *M. truncatula*. **(A)** Phenotype of WT and *mtglk1* mutant plants before and after salt treatment. Scale bar = 5 cm. **(B)** Level of MDA, activity of SOD and POD. Error bars indicate SD (n = 3).

Supplementary Figure 6 Promoter analysis of putative target genes and yeast vector construction. **(A)** Schematic diagrams of MtGLK1-recognition motifs (different type motifs were marked by different color dots) in the promoter regions (2000 bp upstream from ATG) of target genes. **(B)** Schematic representation of the prey vector (*pGADT7-MtGLK1*), in which the *MtGLK1* coding sequence is fused to the GAL4 activation domain (AD). **(C)** Schematic illustrations of the bait vectors, where promoter motifs of *BBX21*, *bHLH25*, and *MYB-HB-like* were cloned upstream of the URA reporter gene in the pABAi vector.

Supplementary Figure 7 Determination of AbA autoactivation thresholds and yeast one-hybrid interaction assay. **(A)** Autoactivation tests for bait vectors containing motifs of *BBX21*, *bHLH1*, and *MYB-HB-like*. The optimal inhibitory concentrations (200 ng/mL for *MYB-HB-like*; 300 ng/mL for *BBX21* and *bHLH25*) were determined for subsequent interaction assays. **(B)** Yeast one-hybrid assays of the interactions between *MtGLK1* and the *BBX21* and *bHLH25* promoter.

Supplementary Figure 8 Expression levels of *MYB-HB-like* in *mtglk1* mutant under salt-alkali stress. **(A)** Expression pattern of *MYB-HB-like* in *mtglk1* mutant under alkali stress for 0, 6, 12, 24 and 48 h. **(B)** Expression pattern of *MYB-HB-like* in *mtglk1* mutant under salt stress for 0, 6, 12, 24 and 48 h. Error bars indicate SD (n = 3). * *P* < 0.05; ** *P* < 0.01.

## References

[B1] Abebe H. Tu Y. (2024). Impact of salt and alkali stress on forage biomass yield, nutritive value, and animal growth performance: a comprehensive review. Grasses 3, 355–368. doi: 10.3390/grasses304002630654563

[B2] Ahmad R. Liu Y. Wang T.-J. Meng Q. Yin H. Wang X. (2019). GOLDEN2-LIKE transcription factors regulate *WRKY40* expression in response to abscisic acid. Plant Physiol. 179 (4), 1844–1860. doi: 10.1104/pp.18.01466 PMC6446771 30723180

[B3] Alam I. Wu X. Yu Q. Ge L. (2022). Comprehensive genomic analysis of G2-like transcription factor genes and their role in development and abiotic stresses in *Arabidopsis*. Diversity 14 (3), 228. doi: 10.3390/d1403022830654563

[B4] Anower R. M. Mott I. W. Peel M. D. Wu Y. (2013). Characterization of physiological responses of two alfalfa half-sib families with improved salt tolerance. Plant Physiol. Biochem. 71, 103–111. doi: 10.1016/j.plaphy.2013.06.026 23911728

[B5] Aouz A. Khan I. Chattha M. B. Ahmad S. Ali M. Ali I. . (2023). Silicon induces heat and salinity tolerance in wheat by increasing antioxidant activities, photosynthetic activity, nutrient homeostasis, and osmo-protectant synthesis. Plants 12, 2606. doi: 10.3390/plants12142606 37514221 PMC10385395

[B6] Bhat B. A. Mir R. A. Mir W. R. Hamdani S. S. Mir M. A. (2024). Transcription factors-golden keys to modulate the plant metabolism to develop salinity tolerance. Plant Stress 11, 10049. doi: 10.1016/j.stress.2024.10040942574925

[B7] Bhattarai S. Biswas D. Fu Y.-B. Biligetu B. (2020). Morphological, physiological, and genetic responses to salt stress in alfalfa: a review. Agronomy 10, 577. doi: 10.3390/agronomy1004057730654563

[B8] Chen H. Zeng Y. Yang Y. Huang L. Tang B. Zhang H. . (2020). Allele-aware chromosome-level genome assembly and efficient transgene-free genome editing for the autotetraploid cultivated alfalfa. Nat. Commun. 11, 2494. doi: 10.1038/s41467-020-16338-x 32427850 PMC7237683

[B9] Clough S. J. Bent A. F. (1998). Floral dip: a simplified method for *Agrocterium*‐mediated transformation of *Arabidopsis thaliana*. Plant J. 16, 735–743. doi: 10.1046/j.1365-313x.1998.00343.x 10069079

[B10] Du J. Lu S. Chai M. Zhou C. Sun L. Tang Y. . (2021). Functional characterization of *PETIOLULE-LIKE PULVINUS (PLP)* gene in abscission zone development in *Medicago truncatula* and its application to genetic improvement of alfalfa. Plant Biotechnol. J. 19 (2), 351–364. doi: 10.1111/pbi.13469 PMC7868985 32816361

[B11] Du W. Yang J. Li Q. Jiang W. Pang Y. (2024). *Medicago truncatula β* glucosidase 17 contributes to drought and salt tolerance through antioxidant flavonoid accumulation. Plant Cell Environ. 47 (8), 3076–3089. doi: 10.1111/pce.14928 38679945

[B12] Fang S. Hou X. Liang X. (2021). Response mechanisms of plants under saline-alkali stress. Front. Plant Sci. 12, 667458. doi: 10.3389/fpls.2021.667458 PMC8213028 34149764

[B13] Fitter D. W. Martin D. J. Copley M. J. Scotland R. W. Langdale J. A. (2002). *GLK* gene pairs regulate chloroplast development in diverse plant species. Plant J. 31, 713–727. doi: 10.1046/j.1365-313X.2002.01390.x 12220263

[B14] Gou J. Debnath S. Sun L. Flanagan A. Tang Y. Jiang Q. . (2017). From model to crop: functional characterization of *SPL8* in *M. truncatula* led to genetic improvement of biomass yield and abiotic stress tolerance in alfalfa. Plant Biotechnol. J. 16 (4), 951–962. doi: 10.1111/pbi.12841 PMC5866946 28941083

[B15] Guo R. Yang Z. Li F. Yan C. Zhong X. Liu Q. . (2015). Comparative metabolic responses and adaptive strategies of wheat (*Triticum aestivum* ) to salt and alkali stress. BMC Plant Biol. 15, 1–13. doi: 10.1186/s12870-015-0546-x 26149720 PMC4492011

[B16] Hall L. N. Rossini L. Cribb L. Langdale J. A. (1998). GOLDEN 2: a novel transcriptional regulator of cellular differentiation in the maize Leaf. Plant Cell 10, 925–936. doi: 10.1105/tpc.10.6.925 9634581 PMC144032

[B17] Hu H. Dai M. Yao J. Xiao B. Li X. Zhang Q. . (2006). Overexpressing a NAM, ATAF, and CUC (NAC) transcription factor enhances drought resistance and salt tolerance in rice. Proc. Natl. Acad. Sci. 103, 12987–12992. doi: 10.1073/pnas.0604882103 16924117 PMC1559740

[B18] Hu G. Li J. Wang X. Kang Y. Li Y. Niu J. . (2023). Molecular evolution and genetic variation of G2-Like transcription factor genes in wheat (*Triticum aestivum* L.). Genes 14 (7), 1341. doi: 10.3390/genes14071341 37510246 PMC10379295

[B19] Huang J. Liu F. Chao D. Xin B. Liu K. Cao S. . (2022). The WRKY transcription factor OsWRKY54 is involved in salt tolerance in rice. Int. J. Mol. Sci. 23, 11999. doi: 10.3390/ijms231911999 36233306 PMC9569829

[B20] Jenkins M. T. (1926). A second gene producing golden plant color in maize. Am. Nat. 60, 484–488. doi: 10.1086/28011939346934

[B21] Jia X. Wang H. Svetla S. Zhu Y. Hu Y. Cheng L. . (2019). Comparative physiological responses and adaptive strategies of apple *Malus halliana* to salt, alkali and saline-alkali stress. Sci. Hortic. 245, 154–162. doi: 10.1016/j.scienta.2018.10.01742574925

[B22] Jiang J. Ma S. Ye N. Jiang M. Cao J. Zhang J. (2017). WRKY transcription factors in plant responses to stresses. J. Integr. Plant Biol. 59, 86–101. doi: 10.1111/jipb.12513 27995748

[B23] Kaur P. Lui C. Dudchenko O. Nandety R. S. Hurgobin B. Pham M. . (2021). Delineating the *Tnt1* Insertion landscape of the model legume *Medicago truncatula* cv. R108 at the Hi-C resolution using a chromosome-length genome assembly. Int. J. Mol. Sci. 22 (9). doi: 10.3390/ijms22094326 PMC8122578 33919286

[B24] Khan I. Mahmood S. Chattha M. U. Chattha M. B. Ahmad S. Awan M. I. (2023). Organic amendments improved the productivity and bio-fortification of fine rice by improving physiological responses and nutrient homeostasis under salinity stress. Plants 12, 1644. doi: 10.3390/plants12081644 37111867 PMC10144191

[B25] Kim G.-B. Nam Y.-W. (2013). A novel Δ1-pyrroline-5-carboxylate synthetase gene of *Medicago truncatula* plays a predominant role in stress-induced proline accumulation during symbiotic nitrogen fixation. J. Plant Physiol. 170 (3), 291–302. doi: 10.1016/j.jplph.2012.10.004 23158502

[B26] Lee K. P. Li M. Li M. Liu K. Medina-Puche L. Qi S. . (2023). Hierarchical regulatory module GENOMES UNCOUPLED1-GOLDEN2-LIKE1/2-WRKY18/40 modulates salicylic acid signaling. Plant Physiol. 192, 3120–3133. doi: 10.1093/plphys/kiad251 37096689

[B27] Li W. Li P. Chen H. Zhong J. Liang X. Wei Y. . (2023). Overexpression of a *Fragaria vesca* 1R-MYB transcription factor gene (*FvMYB114*) increases salt and cold tolerance in *Arabidopsis thaliana*. Int. J. Mol. Sci. 24, 5261. doi: 10.3390/ijms24065261 36982335 PMC10048884

[B28] Li X. Li J. Wei S. Gao Y. Pei H. Geng R. . (2024). Maize GOLDEN2-LIKE proteins enhance drought tolerance in rice by promoting stomatal closure. Plant Physiol. 194, 774–786. doi: 10.1093/plphys/kiad561 37850886 PMC10828204

[B29] Li X. Lin F. Li C. Du L. Liu Z. Shi W. . (2022). *Golden 2-like* transcription factor contributes to the major QTL against rice black-streaked dwarf virus disease. Theor. Appl. Genet. 135, 4233–4243. doi: 10.1007/s00122-022-04214-9 36100693

[B30] Li C. Song T. Zhan L. Cong C. Xu H. Li D. . (2021). Overexpression of MsRCI2A, MsRCI2B, and MsRCI2C in alfalfa (*Medicago sativa* L.) provides different extents of enhanced alkali and salt tolerance due to functional specialization of MsRCI2s. Front. Plant Sci. 12, 702195. doi: 10.3389/fpls.2021.702195 34490005 PMC8417119

[B31] Liu Z. Chen T. Ma L. Zhao Z. Zhao P. X. Nan Z. . (2013). Global transcriptome sequencing using the Illumina platform and the development of EST-SSR markers in autotetraploid alfalfa. PloS One 8, e83549. doi: 10.1371/journal.pone.0083549 24349529 PMC3861513

[B32] Liu X. Li L. Li M. Su L. Lian S. Zhang B. . (2018). *AhGLK1* affects chlorophyll biosynthesis and photosynthesis in peanut leaves during recovery from drought. Sci. Rep. 8 (1), 2250. doi: 10.1038/s41598-018-20542-7 29396501 PMC5796971

[B33] Liu Z. Li J. Li S. Song Q. Miao M. Fan T. . (2025). The 1R-MYB transcription factor SlMYB1L modulates drought tolerance via an ABA-dependent pathway in tomato. Plant Physiol. Biochem. 222, 109721. doi: 10.1016/j.plaphy.2025.109721 40056740

[B34] Liu L. Li S. Tang F. Li P. Liu J. Fu R. . (2024). MaMYBR30, a novel 1R-MYB, plays important roles in plant development and abiotic stress resistance. Plants 13, 1794. doi: 10.3390/plants13131794 38999634 PMC11244220

[B35] Liu W. Zhang Z. Chen S. Ma L. Wang H. Dong R. . (2020). AhHDA1-mediated *AhGLK1* promoted chlorophyll synthesis and photosynthesis regulates recovery growth of peanut leaves after water stress. Plant Sci. 294, 110461. doi: 10.1016/j.plantsci.2020.110461 32234234

[B36] Liu J. Mehari T. G. Xu Y. Umer M. J. Hou Y. Wang Y. . (2021). *GhGLK1* a key candidate gene from GARP family enhances cold and drought stress tolerance in cotton. Front. Plant Sci. 12. doi: 10.3389/fpls.2021.759312 34992618 PMC8725998

[B37] Liu W. Zhang Z. Chen S. Ma L. Wang H. Dong R. . (2016). Global transcriptome profiling analysis reveals insight into saliva-responsive genes in alfalfa. Plant Cell Rep. 35, 561–571. doi: 10.1007/s00299-015-1903-9 26645698

[B38] Lu J. Shen Y. Yang Y. Li X. Li X. Liu D. . (2023). Synthetic strigolactone (*rac* GR24) alleviates the photosynthetic inhibition and oxidative damage in alfalfa (*Medicago sativa* L.) under salt stress. Grassl. Sci. 70 (1), 23–34. doi: 10.1111/grs.12417

[B39] Luo D. Liu J. Wu Y. Zhang X. Zhou Q. Fang L. . (2022). NUCLEAR TRANSPORT FACTOR 2‐LIKE improves drought tolerance by modulating leaf water loss in alfalfa (*Medicago sativa* L.). Plant J. 112, 429–450. doi: 10.1111/tpj.15955 36006043

[B40] Luo D. Zhang X. Li Y. Wu Y. Li P. Jia C. . (2023). *MsDIUP1* encoding a putative novel LEA protein positively modulates salt tolerance in alfalfa (*Medicago sativa* L.). Plant Soil 487, 547–566. doi: 10.1007/s11104-023-05951-630311153

[B41] Miller M. Song Q. Shi X. Juenger T. E. Chen Z. J. (2015). Natural variation in timing of stress-responsive gene expression predicts heterosis in intraspecific hybrids of *Arabidopsis*. Nat. Commun. 6, 7453. doi: 10.1038/ncomms8453 26154604

[B42] Min X. Luo K. Liu W. Zhou K. Li J. Wei Z. (2022). Molecular characterization of the miR156/MsSPL model in regulating the compound leaf development and abiotic stress response in alfalfa. Genes 13 (2). doi: 10.3390/genes13020331 PMC8871590 35205375

[B43] Murmu J. Wilton M. Allard G. Pandeya R. Desveaux D. Singh J. . (2013). *Arabidopsis* GOLDEN2‐LIKE (GLK) transcription factors activate jasmonic acid (JA)‐dependent disease susceptibility to the biotrophic pathogen *Hyaloperonospora arabidopsidis*, as well as JA‐independent plant immunity against the necrotrophic pathogen *Botrytis cinerea*. Mol. Plant Pathol. 15, 174–194. doi: 10.1111/mpp.12077 24393452 PMC6638812

[B44] Nagatoshi Y. Mitsuda N. Hayashi M. Inoue S.-i. Okuma E. Kubo A. . (2016). *GOLDEN 2-LIKE* transcription factors for chloroplast development affect ozone tolerance through the regulation of stomatal movement. Proc. Natl. Acad. Sci. 113, 4218–4223. doi: 10.1073/pnas.1513093113 27035938 PMC4839443

[B45] Nguyen C. V. Vrebalov J. T. Gapper N. E. Zheng Y. Zhong S. Fei Z. . (2014). Tomato *GOLDEN2-LIKE* transcription factors reveal molecular gradients that function during fruit development and ripening. Plant Cell 26, 585–601. doi: 10.1105/tpc.113.118794 24510723 PMC3967027

[B46] Nguyen M. L. Kim G.-B. Hyun S.-H. Lee S.-Y. Lee C.-Y. Choi H.-K. . (2013). Physiological and metabolomic analysis of a knockout mutant suggests a critical role of *MtP5CS3* gene in osmotic stress tolerance of *Medicago truncatula*. Euphytica 193 (1), 101–120. doi: 10.1007/s10681-013-0957-4

[B47] Orellana S. Yanez M. Espinoza A. Verdugo I. Gonzalez E. Ruiz-Lara S. . (2010). The transcription factor SlAREB1 confers drought, salt stress tolerance and regulates biotic and abiotic stress‐related genes in tomato. Plant Cell Environ. 33, 2191–2208. doi: 10.1111/j.1365-3040.2010.02220.x 20807374

[B48] Powell A. L. T. V. N. C. H. Theresa Lam C. K. Rosa F.-B. Hakan A. . (2012). *Uniform* ripening encodes a *Golden 2-like* transcription factor regulating tomato fruit chloroplast development. Science 336, 1711–1718. doi: 10.1126/science.1222218 22745430

[B49] Puranik S. Sahu P. P. Srivastava P. S. Prasad M. (2012). NAC proteins: regulation and role in stress tolerance. Trends Plant Sci. 17, 369–381. doi: 10.1016/j.tplants.2012.02.004 22445067

[B50] Ren Y. Liu L. Zhao W. Guo W. Li J. Guo R. . (2026). MsCDPK29-MsbZIP14 modulates ABA accumulation in response to saline-alkali stress in alfalfa. New Phytol. 250, 332–346. doi: 10.1111/nph.70945 41574443

[B51] Rossini L. Cribb L. Martin D. J. Langdale J. A. (2001). The maize *golden2* gene defines a novel class of transcriptional regulators in plants. Plant Cell 13, 1231–1244. doi: 10.1105/tpc.13.5.1231 11340194 PMC135554

[B52] Safavi-Rizi V. Friedlein H. Safavi-Rizi S. Krajinski-Barth F. (2025). The impact of arbuscular mycorrhizal colonization on flooding response of *Medicago truncatula*. Front. Plant Sci. 15. doi: 10.3389/fpls.2024.1512350 PMC11750877 39845493

[B53] Shi D. Sheng Y. (2005). Effect of various salt–alkaline mixed stress conditions on sunflower seedlings and analysis of their stress factors. Environ. Exp. Bot. 54, 8–21. doi: 10.1016/j.envexpbot.2004.05.00342574925

[B54] Shi D. Wang D. (2005). Effects of various salt-alkaline mixed stresses on *Aneurolepidium chinense* (Trin.) Kitag. Plant Soil 271, 15–26. doi: 10.1007/s11104-004-1307-z30311153

[B55] Sun N. Song T. Ma Z. Dong L. Zhan L. Xing Y. . (2020). Overexpression of *MsSiR* enhances alkali tolerance in alfalfa (*Medicago sativa* L.) by increasing the glutathione content. Plant Physiol. Biochem. 154, 538–546. doi: 10.1016/j.plaphy.2020.07.001 32912487

[B56] Sun Q. Huang R. Zhu H. Sun Y. Guo Z. (2021). A novel *Medicago truncatula* calmodulin like protein (MtCML42) regulates cold tolerance and flowering time. Plant J. 108 (4), 1069–1082. doi: 10.1111/tpj.15494 34528312

[B57] Tadege M. Ratet P. Mysore K. S. (2005). Insertional mutagenesis: a Swiss Army knife for functional genomics of *Medicago truncatula*. Trends Plant Sci. 10 (5), 229–235. doi: 10.1016/j.tplants.2005.03.009 15882655

[B58] Tadege M. Wen J. He J. Tu H. Kwak Y. Eschstruth A. . (2008). Large scale insertional mutagenesis using the *Tnt1* retrotransposon in the model legume *Medicago truncatula*. Plant J. 54 (2), 335–347. doi: 10.1111/j.1365-313X.2008.03418.x 18208518

[B59] Tang H. Krishnakumar V. Bidwell S. Rosen B. Chan A. Zhou S. . (2014). An improved genome release (version Mt4. 0) for the model legume *Medicago truncatula*. BMC Genomics 15, 1–14. doi: 10.1186/1471-2164-15-312 24767513 PMC4234490

[B60] Townsend P. D. Dixon C. H. Slootweg E. J. Sukarta O. C. A. Yang A. W. H. Hughes T. R. . (2018). The intracellular immune receptor Rx1 regulates the DNA-binding activity of a Golden2-LIKE transcription factor. J. Biol. Chem. 293, 3218–3233. doi: 10.1074/jbc.RA117.000485 29217772 PMC5836133

[B61] Wan S. Zhang Y. Liu L. Xiao Y. He J. Zhang Y. . (2024). Comparative effects of salt and alkali stress on photosynthesis and antioxidant system in tea plant (*Camellia sinensis*). Plant Growth Regul. 103, 565–579. doi: 10.1007/s10725-023-01115-930311153

[B62] Wang X. Chen W. Zhou Y. Han J. Zhao J. Shi D. . (2012). Comparison of adaptive strategies of alfalfa (*Medicago sativa* L.) to salt and alkali stresses. Aust. J. Crop Sci. 6, 309–315. doi: 10.3316/informit.054677379876569

[B63] Wang X. Geng S. Ri Y.-J. Cao D. Liu J. Shi D. . (2011). Physiological responses and adaptive strategies of tomato plants to salt and alkali stresses. Sci. Hortic. 130, 248–255. doi: 10.1016/j.scienta.2011.07.00642574925

[B64] Wang J. Li Q. Zhang M. Wang Y. (2022). The high pH value of alkaline salt destroys the root membrane permeability of *Reaumuria trigyna* and leads to its serious physiological decline. J. Plant Res. 135, 785–798. doi: 10.1007/s10265-022-01410-y 36266589

[B65] Wang X. Ren H. Wei Z. Wang Y. Ren W. (2017). Effects of neutral salt and alkali on ion distributions in the roots, shoots, and leaves of two alfalfa cultivars with differing degrees of salt tolerance. J. Integr. Agric. 16, 1800–1807. doi: 10.1016/S2095-3119(16)61522-8

[B66] Waters M. T. Wang P. Korkaric M. Capper R. G. Saunders N. J. Langdale J. A. (2009). *GLK* transcription factors coordinate expression of the photosynthetic apparatus in *Arabidopsis*. Plant Cell 21, 1109–1128. doi: 10.1105/tpc.108.065250 19376934 PMC2685620

[B67] Wong C. E. Li Y. Labbe A. Guevara D. Nuin P. Whitty B. . (2006). Transcriptional profiling implicates novel interactions between abiotic stress and hormonal responses in *Thellungiella*, a close relative of *Arabidopsis*. Plant Physiol. 140, 1437–1450. doi: 10.1104/pp.105.070508 16500996 PMC1435811

[B68] Wu J. Jiang Y. Liang Y. Chen L. Chen W. Cheng B. (2019). Expression of the maize MYB transcription factor ZmMYB3R enhances drought and salt stress tolerance in transgenic plants. Plant Physiol. Biochem. 137, 179–188. doi: 10.1016/j.plaphy.2019.02.010 30798172

[B69] Xu Z. S. Chen M. Li L. C. Ma Y. Z. (2011). Functions and application of the AP2/ERF transcription factor family in crop improvement. J. Integr. Plant Biol. 53, 570–585. doi: 10.1111/j.1744-7909.2011.01062.x 21676172

[B70] Yamaguchi-Shinozaki K. Shinozaki K. (2006). Transcriptional regulatory networks in cellular responses and tolerance to dehydration and cold stresses. Annu. Rev. Plant Biol. 57, 781–803. doi: 10.1146/annurev.arplant.57.032905.105444 16669782

[B71] Yasumura Y. Moylan E. C. Langdale J. A. (2005). A conserved transcription factor mediates nuclear control of organelle biogenesis in anciently diverged land plants. Plant Cell 17, 1894–1907. doi: 10.1105/tpc.105.033191 15923345 PMC1167540

[B72] Yoo J. H. Park C. Y. Kim J. C. Do Heo W. Cheong M. S. Park H. C. . (2005). Direct interaction of a divergent CaM isoform and the transcription factor, MYB2, enhances salt tolerance in *Arabidopsis*. J. Biol. Chem. 280, 3697–3706. doi: 10.1074/jbc.M408237200 15569682

[B73] Young N. D. Debellé F. Oldroyd G. E. D. Geurts R. Cannon S. B. Udvardi M. K. . (2011). The *Medicago* genome provides insight into the evolution of rhizobial symbioses. Nature 480 (7378), 520–524. doi: 10.1038/nature10625 PMC3272368 22089132

[B74] Yu J. Yuan Y. Zhang W. Song T. Hou X. Kong L. . (2022). Overexpression of an *NF-YC2* gene confers alkali tolerance to transgenic alfalfa (*Medicago sativa* L.). Front. Plant Sci. 13, 960160. doi: 10.3389/fpls.2022.960160 35991397 PMC9389336

[B75] Yuan Y. Song T. Yu J. Zhang W. Hou X. Kong Ling Z. . (2022). Genome-wide investigation of the cysteine synthase gene family shows that overexpression of *CSase* confers alkali tolerance to alfalfa (*Medicago sativa* L.). Front. Plant Sci. 12. doi: 10.3389/fpls.2021.792862 35058952 PMC8765340

[B76] Zhang H. Li X. Che Y. Wang Y. Li M. Yang R. . (2020a). A study on the effects of salinity and pH on PSII function in mulberry seedling leaves under saline–alkali mixed stress. Trees 34, 693–706. doi: 10.1007/s00468-019-01949-930311153

[B77] Zhang D. Shen Z. He P. Wang J. Li D. Meng J. . (2025). The synergistic roles of MsRCI2B and MsRCI2E in the regulation of ion balance and ROS homeostasis in alfalfa under salt stress. Int. J. Biol. Macromol. 300. doi: 10.1016/j.ijbiomac.2025.140093 39863229

[B78] Zhang W. Wang N. Yang J. Guo H. Liu Z. Zheng X. . (2020b). The salt-induced transcription factor GmMYB84 confers salinity tolerance in soybean. Plant Sci. 291, 110326. doi: 10.1016/j.plantsci.2019.110326 31928665

[B79] Zhang H. Yao T. Zhang H. Zhang Z. Wang K. Qi S. . (2024). Hormonal signaling regulates photosynthetic function of alfalfa (*Medicago sativa* L.) under NaHCO_3_ stress. Environ. Exp. Bot. 228. doi: 10.1016/j.envexpbot.2024.10599942574925

[B80] Zhao Z. Chai M. Sun L. Cong Jiang Q. Zhang . (2021). Identification of a gene responsible for seedpod spine formation and other phenotypic alterations using whole-genome sequencing analysis in *Medicago truncatula*. J. Exp. Bot. 72 (22), 7769–7777. doi: 10.1093/jxb/erab359 34329408

[B81] Zheng M. Wang X. Luo J. Ma B. Li D. Chen X. (2024). The pleiotropic functions of GOLDEN2-LIKE transcription factors in plants. Front. Plant Sci. 15. doi: 10.3389/fpls.2024.1445875 39224848 PMC11366661

[B82] Zhou C. Han L. Li G. Chai Fu C. Cheng X. . (2014). STM/BP-Like KNOXI is uncoupled from ARP in the regulation of compound leaf development in *Medicago truncatula*. Plant Cell 26 (4), 1464–1479. doi: 10.1105/tpc.114.123885 PMC4036565 24781113

[B83] Zhou C. Han L. Hou C. Metelli A. Qi L. Tadege M. . (2011). Developmental analysis of a *Medicago truncatula smooth leaf margin1* mutant reveals context-dependent effects on compound leaf development. Plant Cell 23 (6), 2106–2124. doi: 10.1105/tpc.111.085464 PMC3160044 21693694

[B84] Zhu J.-K. (2001). Plant salt tolerance. Trends Plant Sci. 6, 66–71. doi: 10.1016/S1360-1385(00)01838-0 11173290

